# Malignant transformation of oral leukoplakia: Systematic review and comprehensive meta‐analysis

**DOI:** 10.1111/odi.15140

**Published:** 2024-09-24

**Authors:** Liliana Aparecida Pimenta‐Barros, Pablo Ramos‐García, Miguel Ángel González‐Moles, José Manuel Aguirre‐Urizar, Saman Warnakulasuriya

**Affiliations:** ^1^ Dental Sciences Graduate Program Federal University of Espirito Santo Vitoria Brazil; ^2^ Instituto de Investigación Biosanitaria, School of Dentistry University of Granada Granada Spain; ^3^ Oral and Maxillofacial Medicine & Pathology Unit, Department of Stomatology, Faculty of Medicine and Nursery University of the Basque Country/EHU Leioa Spain; ^4^ Faculty of Dental, Oral & Craniofacial Sciences WHO Collaborating Centre for Oral Cancer, King's College London London UK

**Keywords:** malignant transformation, meta‐analysis, oral cancer, oral leukoplakia, oral potentially malignant disorder, systematic review

## Abstract

**Objectives:**

To update the current evidence on the malignant transformation of oral leukoplakia (OL), including all studies published worldwide on the subject, selected with the maximum rigor regarding eligibility.

**Materials and Methods:**

MEDLINE, Embase, Web of Science and Scopus were searched for studies published before June‐2024, with no lower date limit. The risk of bias was analyzed using the Joanna Briggs Institute tool for meta‐analyses of proportions. We carried out meta‐analyses, explored heterogeneity across subgroups and identified risk factors with potential prognostic value.

**Results:**

Fifty‐five studies (41,231 with OL) were included. The pooled malignant transformation proportion for OL was 6.64% (95% CI = 5.21–8.21). The malignant transformation did not significantly vary by time periods (*p* = 0.75), 5.35% prior to 1978, 7.06% from 1979 to 2007 and 6.97% during more recent times. The risk factors that significantly had a higher impact on malignant transformation were the non‐homogeneous leukoplakias (RR = 4.23, 95% CI = 3.31–5.39, *p* < 0.001), the larger size (RR = 2.08, 1.45–2.96, *p* < 0.001), leukoplakia located on the lateral border of tongue (malignant transformation = 12.71%; RR = 2.09, 95% CI = 1.48–2.95, *p* < 0.001), smoking (RR = 1.64, 95% CI = 1.25–2.15, *p* < 0.001), and the presence of epithelial dysplasia (RR = 2.75, 95% CI = 2.26–3.35, *p* < 0.001).

**Conclusions:**

OL presents a considerable malignant transformation probability that is especially increased in large non‐homogeneous lesions in smokers, located on the lateral border of the tongue, with epithelial dysplasia.

## 1 INTRODUCTION

Oral leukoplakia (OL) is the most important oral potentially malignant disorder (OPMD) affecting the oral mucosa. It is considered a common condition with a worldwide prevalence ranging from 1.36% to 2.60% in the general population according to the three published systematic reviews and meta‐analyses (Mello et al., 2018; Petti, 2003; Zhang et al., 2023). At the last international seminar on the nomenclature and classification of OPMDs (Glasgow, Scotland in March 2020) convened by the WHO Collaborating Centre for Oral Cancer (UK), an international group of experts defined OL as a predominantly white plaque of questionable risk having excluded (other) known diseases or disorders that carry no increased risk for cancer (Warnakulasuriya et al., 2021). Since OL was described by Schwimmer (1877) and identified as premalignant in 1925 Fox (1925), several articles have been published in the international scientific literature concerning its potential malignancy, the probability of transformation and describing the risk factors affecting its progression to oral squamous cell carcinoma. Data derived from primary‐level studies (case series with follow up data) give widely varying figures for malignancy of OL ranging from 0.09% to 38.5% of cases (Farah et al., 2019; Gupta et al., 1980). To date, four systematic reviews and meta‐analyses have been published on the development of cancer in OL (Aguirre‐Urizar et al., 2021; Guan et al., 2023; Iocca et al., 2020; Warnakulasuriya & Ariyawardana, 2016) that show malignancy rates ranging from 7.20% to 9.80%, none of them completely covering all the primary‐level studies reported since 1934 that appear in the international scientific literature.

A definition for OL was first published in a report commissioned by the WHO in 1978 (Kramer et al., 1978) and was modified in 2005 by an expert group of the WHO Collaborating Centre (published in 2007) (Warnakulasuriya et al., 2007). The criteria for the detection of OL therefore varied in these time periods. In this meta‐analysis, we therefore propose to stratify data by these time periods. The aim of this systematic review and meta‐analysis was to comprehensively cover the total time period to date since the description of the first case of oral leukoplakia, to analyze whether the rate of malignant transformation has changed over time and according to the different concepts that have been proposed for this lesion, and to explore the clinicopathological factors that determine the malignant transformation.

## 2 MATERIALS AND METHODS

This systematic review and meta‐analysis was designed and conducted in accordance with *Cochrane Collaboration* (Higgins & Green, 2008) and *Joanna Briggs Institute* (Aromataris et al., 2024) methodological criteria for evidence synthesis. The reporting of this manuscript strictly complies with PRISMA and MOOSE reporting guidelines (Page et al., 2021; Stroup et al., 2000).

### 2.1 Protocol

In order to reduce bias and ensure transparency, accuracy and integrity, a methodology protocol was pre‐registered with the international prospective systematic review register PROSPERO (www.crd.york.ac.uk/PROSPERO; registration number: ID‐574224/CRD42024574224) (Booth et al., 2012). The protocol also adheres to the PRISMA‐P guidelines, guaranteeing a rigorous approach (Shamseer et al., 2015).

### 2.2 Search strategy

A comprehensive search was conducted in the MEDLINE (via PubMed), Embase, Web of Science, and Scopus databases, covering databases published up to June 2024. This search was conducted by combining thesaurus terms used by the databases (i.e., MeSH and Emtree) with free terms, and designed with the purpose to maximize sensitivity. The search strategy combined the terms “oral leukoplakia,” “malignant transformation” and synonyms (the full syntax applied to all databases is available as Table S1, in the supplementary information). In addition, the reference lists of retrieved studies were manually screened for the inclusion of further relevant studies. All references were managed using Mendeley 1.19.8 (Elsevier, Amsterdam, the Netherlands), and duplicate references were excluded also using this software.

### 2.3 Eligibility criteria

Following the Condition, Context and Population *CoCoPop* framework – specifically designed from Joanna Briggs Institute (University of Adelaide, Australia) for systematic reviews/meta‐analyses of proportions (Aromataris et al., 2024) – the following scientific framework was investigated: Studies investigating the proportion of cases with malignant transformation (condition); in individuals with OL (population); and their related characteristics clinicopathological characteristics, assessed through cohorts of follow‐up studies, without restrictions by publication language or date (context). Therefore, the following inclusion criteria were applied: (1) Original primary‐level studies published reporting the malignant transformation probability of OL; (2) Observational epidemiological studies designed as longitudinal cohorts; (3) No restrictions by language, publication date, length of follow‐up periods, geographical area, age or sex.

The following exclusion criteria were applied: (1) Retracted articles, reviews, meta‐analyses, case reports, editorials, letters, abstracts from scientific meetings, personal opinions, comments or book chapters; (2) Studies conducted on in vitro or animal models; (3) Different epidemiological study design (i.e., interventionist, case–control studies or cross‐sectional); (4) RCTs that had any intervention to alter the natural history; (5) Studies that do not analyze the malignant transformation of OL, or the failure to provide sufficient data for its calculation; (6) studies investigating other types of oral potentially malignant disorders (e.g., proliferative verrucous leukoplakia, oral lichen planus or oral submucous fibrosis) or not reporting separate data for OL; (7) Studies that do not differentiate between lesions from other anatomical sites; (8) Overlapping population studies. In case of overlapping studies on the same population, the latest publication or the more informative was selected for inclusion.

Articles were selected in two phases by two researchers (LAPB and PRG): first phase, screening of titles and abstracts in order to include those articles apparently meeting eligibility criteria; second phase, reading of full‐text articles, and excluding those that failed to meet the eligibility criteria.

### 2.4 Data extraction

Upon a thorough full‐text examination, one author (LAPB) extracted data from the selected articles using a standardized data collection form in Excel (v. 16/2018, Microsoft, Redmond, WA). The extracted datasets were additionally cross‐checked in a collaborative manner by a second author (PRG), resolving discrepancies by consensus. Using the methods proposed by Luo et al. (2018) and Wan et al. (2014), data expressed as median, interquartile range and/or maximum‐minimum values were calculated and converted to means‐standard deviation (SD) when possible. In cases where it was desirable to combine two or more different datasets expressed as means‐standard deviation of subgroups into a single group, the Cochrane Handbook formula was applied (Higgins & Green, 2008). The datasets gathered information on the study (first author), year, country and continent, study design, anatomical sites affected, size and clinical appearance of lesions, number of patients with OL, malignant transformation proportion, time to malignant transformation, follow‐up and recruitment periods, sex and age of patients, tobacco, betel quid and alcohol use, presence and grade of oral epithelial dysplasia.

### 2.5 Evaluation of quality and risk of bias

The quality of primary‐level studies was critically judged by one author (LAPB), evaluated using a specific method designed for systematic reviews/meta‐analyses of proportions (Joanna Briggs Institute, University of Adelaide, Australia) (Aromataris et al., 2024). Risk of bias was evaluated for each individual study across the following domains: (1) Was the sample frame appropriate to address the target population? (2) Were study participants sampled in an appropriate way? (3) Was the sample size adequate? (4) Were the study subjects and the setting described in detail? (5) Was the data analysis conducted with sufficient coverage of the identified sample? (6) Were valid methods used for the identification of the condition? (7) Was the condition measured in a standard, reliable way for all participants? (8) Was there appropriate statistical analysis? (9) Was the response rate adequate, and if not, was the low response rate managed appropriately? Each domain was then scored as “Yes” (low risk of bias), “Unclear” (moderate risk) or “No” (High risk).

### 2.6 Statistical analysis

The malignant transformation of OL was estimated through meta‐analytical techniques by combining proportions with 95% confidence intervals (CIs). These proportions were calculated by extracting raw numerators (total number of malignant transformation cases) and denominators (total number of patients with OL), and 95% Cis were computed according to the Wilson's score method (Agresti & Coull, 1998). The influence of studies with extremely small values was minimized by implementing Freeman‐Tukey double‐arcsine transformations to stabilize the variance of proportions (Freeman & Tuckey, 1950), with the subsequent back‐transformation of the pooled proportions (Nyaga et al., 2014). All meta‐analyses of proportions were carried out under a random‐effects model (based on the DerSimonian and Laird method) (DerSimonian & Laird, 1986). Furthermore, several additional meta‐analyses were performed in order to quantitatively evaluate the magnitude of association between risk factors and the development of oral cancer. For this purpose, relative risks (RR) with 95% CIs were pooled using the Mantel–Haenszel method (fixed‐effect model), due to potentially better statistical properties. Forest plots were constructed for all meta‐analyses, in order to graphically represent the effect sizes and for subsequent visual inspection analysis. The *χ* ^2^‐based Cochran's *Q* test was used to assess the between‐study heterogeneity (Higgins & Thompson, 2002); given its low statistical power, *p* < 0.1 was considered significant, assuming apparent statistical heterogeneity. The Higgins *I* ^2^ statistic was also used to quantify the percentage heterogeneity, with results of 25%–50%–75% indicating, respectively, low, moderate, and high heterogeneity (Higgins et al., 2003; Higgins & Thompson, 2002). Stratified meta‐analyses were performed in order to identify potential sources of heterogeneity (Borenstein & Higgins, 2013). Sensitivity analyses were also carried out, in order to assess the influence of individual primary‐level studies on the overall pooled malignant transformation proportion (Viechtbauer & Cheung, 2010). For this purpose, the meta‐analysis was repeated, omitting one study at a time (i.e., the so‐called leave‐one‐out method). Finally, funnel plots were constructed and the Egger regression test (*p*_Egger_ <0.1) was applied to evaluate small‐study effects such as publication bias (Egger et al., 1997; Jin et al., 2015; Sterne et al., 2011). Stata software was used for all statistical analyses (v.16.1, Stata Corp, College Station, TX, USA).

## 3 RESULTS

### 3.1 Literature search

**FIGURE 1:**
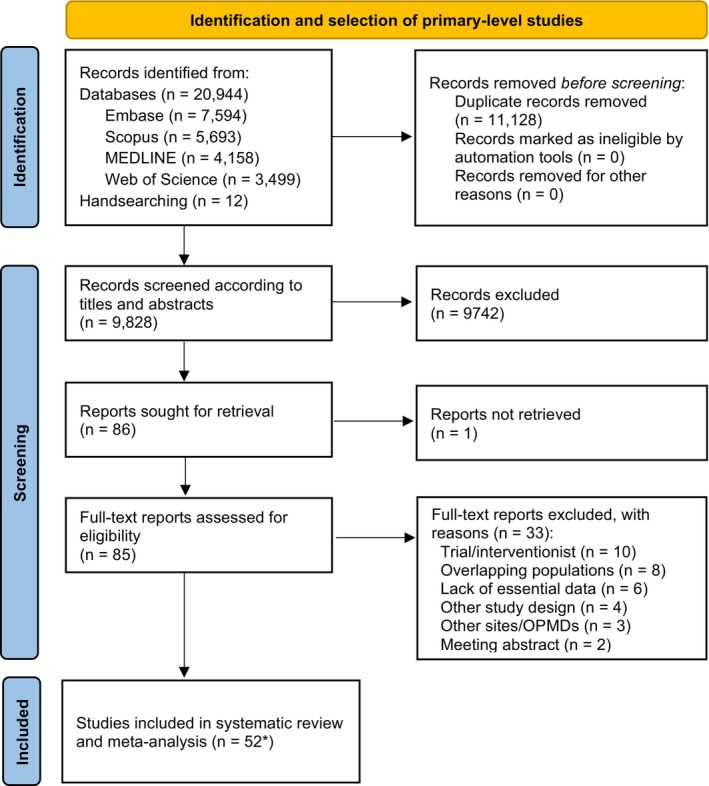
Flow diagram of the process of identification and selection of primary‐level studies offering scientific information on the malignant transformation of oral leukoplakias. *Fifty two published studies that included 55 different units of analysis.

The flow diagram (Figure 1) depicts the results of the literature search and the study selection process. We retrieved a total of 20,944 records published before June 2024: 7594 from Embase, 5593 from Scopus, 4158 from MEDLINE (through PubMed), 3499 from Web of Science, and 12 from other sources by applying handsearching methods. After removal of duplicates, 9828 studies were considered potentially eligible and screened for titles and abstracts. Subsequently, 85 full‐text reports were retrieved, of which 33 did not meet all inclusion criteria, leaving a final sample of 52 studies (the references of the studies included and excluded – with reasons – are available in the supplementary information, Lists S1 and S2). Finally, 52 published studies were systematically reviewed and meta‐analyzed as 55 different units of analysis (and considered as separate studies throughout the manuscript).

### 3.2 Study characteristics

Table 1 and Table S2 in the supplementary information exhibit the characteristics and variables gathered. The 55 studies that were selected recruited 41,231 study subjects (range: 13–6718), of whom 1575 OL transformed in oral cancer. Twenty studies were conducted in Asia, 25 in Europe, 5 in North America, 3 in South America, and 2 in Oceania. According to the clinical settings, 12 were population‐based and 43 were clinic‐based studies. Prior to 1978, 12 studies were published, 13 studies from 1979 to 2007 and 30 studies after 2007. Thirty‐two studies reported sufficient data on sex to be included in the quantitative analysis of this parameter, with a study population of 19,657 males and 4299 females. In relation to age, only six studies reported data for quantitative analysis, including a study population of 1922 patients over 50 years old. Furthermore, 12 and 8 studies reported, respectively, data on tobacco and alcohol consumption to be included in the quantitative analyses of these parameters, with study populations of 9030 smokers and 1027 alcohol drinkers.

**TABLE 1:** Summarized characteristics of reviewed studies.

Total	55 studies^a^
Year of publication	1934–2023
Number of patients	
Total	41,231
Developing oral cancer	1575
Sample size, range	13–6718 patients
Source of patients	
Population‐based studies	12 studies
Clinic‐based studies	43 studies
Study design	
Retrospective longitudinal	47 studies
Prospective longitudinal	8 studies
Geographical region	
Europe	25 studies
Asia	20 studies
North America	5 studies
South America	3 studies
Oceania	2 studies

*Note*: Table S2 (supplementary information) summarizes the characteristics of each study.

^a^ Fifty two published studies that included 55 different units of analysis.

### 3.3 Qualitative evaluation

**FIGURE 2:**
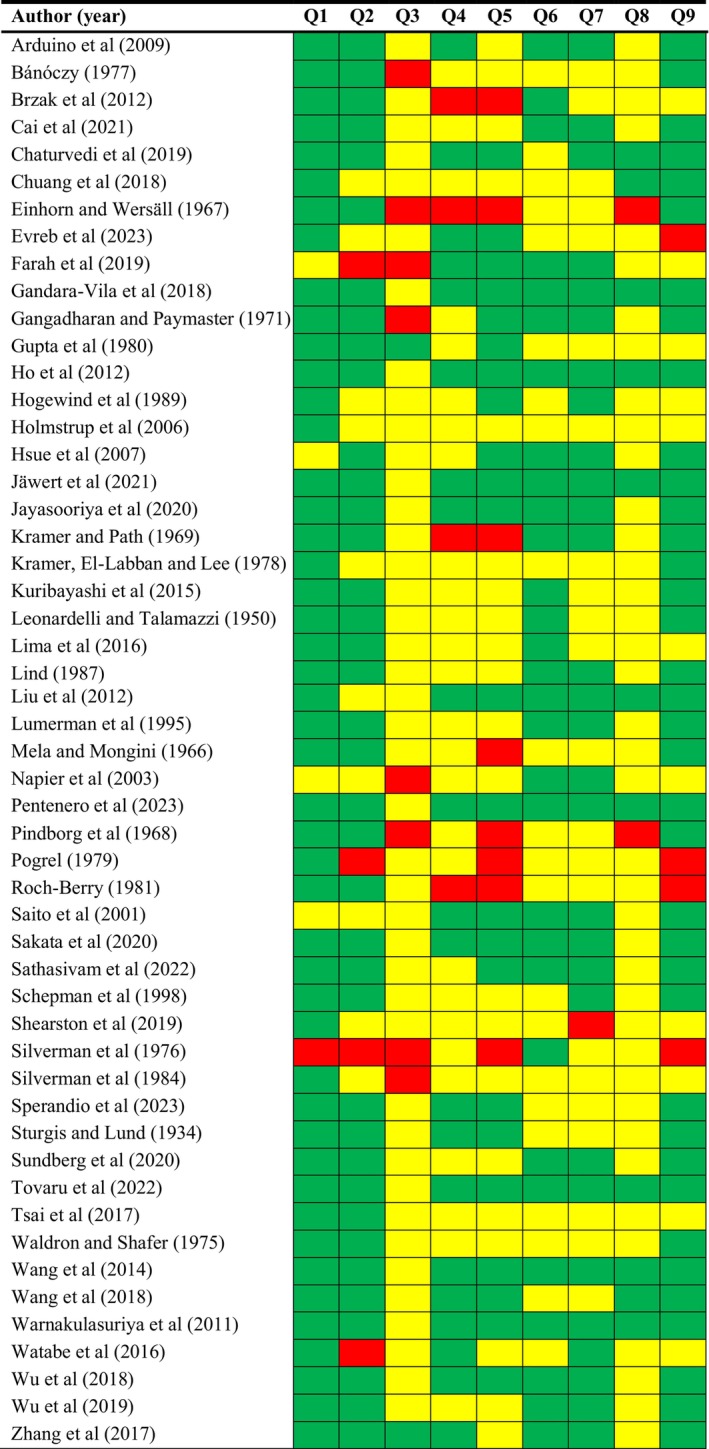
Quality plot graphically representing the risk of bias across primary‐level studies, critically appraising ten domains, using a method specifically designed for meta‐analyses of proportions (developed by the Joanna Briggs Institute, University of Adelaide, South Australia). Green, low risk of potential bias; yellow, moderate; red, high.

After the methodological quality and risk of bias analysis was performed across primary‐level studies, in a global view, the majority of studies were not conducted with the same rigor in accordance with the specific Joanna Briggs Institute tool (Figure 2). However, in most studies, samples were appropriate to address the target population (Q1) and recruited in an appropriate way (Q2). The highest risk of potential bias was displayed by items Q3, Q4 and Q8. Regarding domain Q3, there was a lack of application of sampling methods in most of the primary‐level studies (i.e., random recruitment methods, statistical calculation of sample size). Concerning domain Q4, primary‐level studies failed to communicate potentially confounding variables and offer more details of the sample of OL (i.e., age, alcohol or tobacco or betel consumption, size, clinical type and oral epithelial dysplasia) and finally, domain Q8 reported insufficient or not appropriate statistical analysis.

### 3.4 Quantitative evaluation (meta‐analyses)

**FIGURE 3:**
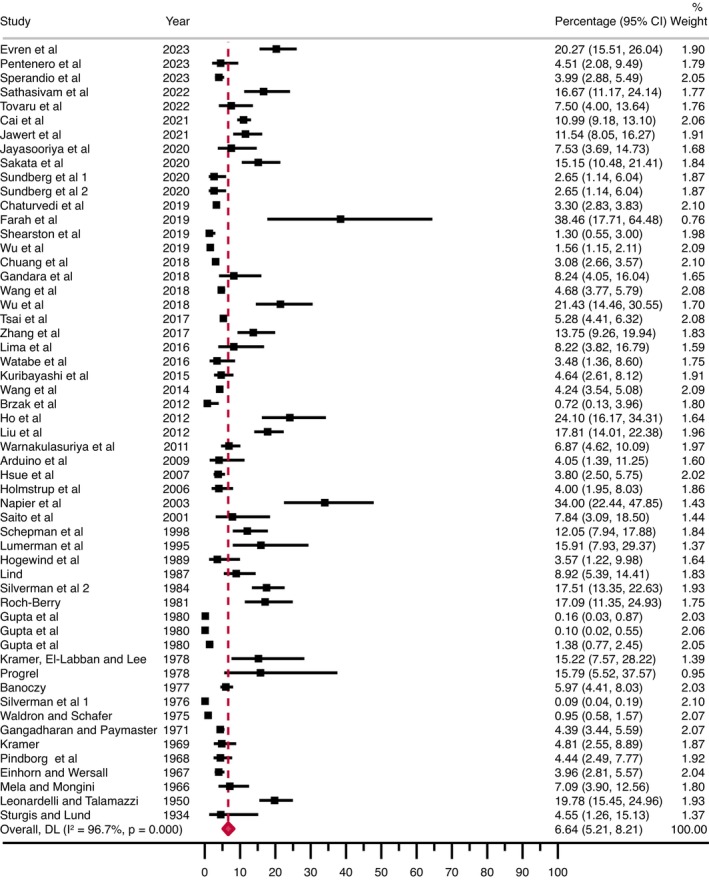
Forest plot graphically representing the meta‐analysis of the malignant transformation proportion of oral leukoplakia. Pooled proportions (expressed as percentage) were used as effect size metric, jointly with their corresponding 95% confidence intervals (CI).

In the main meta‐analysis, the malignant transformation pooled proportion for OL was 6.64% (95% CI = 5.21–8.21; *p*_het_ <0.001, *I* ^2^ = 96.7%), as shown in Figure 3. Multiple additional meta‐analyses were run in order to evaluate the malignant transformation of OL across subgroups and risk factors with potential prognostic value (Table 2, Figures S1–S28 in supplementary information).

**TABLE 2:** Malignant transformation of oral leukoplakia and related variables.

Analyses	No. of studies	No. of patients	Stat. model	Pooled data			
				ES (95% CI)	*p*‐value	*p*_*het*_	*I* ^*2*^ (%)
Malignant transformation^b^ (overall pooled proportion)	55^a^	41,231	R, d‐l	PP = 6.64% (5.21–8.21)		<0.001	96.7
Source of patients^c^							
Population‐based study	12	12,514	R, d‐l	PP = 3.10% (1.50–5.22)	<0.001^e^	<0.001	98.7
Clinic‐based study	43	28,717	R, d‐l	PP = 8.06% (6.23–10.08)		<0.001	92.4
Geographical región^c^							
Asia	20	28,037	R, d‐l	PP = 4.88% (3.04–7.10)	0.03^e^	<0.001	98.1
Europe	25	4850	R, d‐l	PP = 8.94% (6.54–11.64)		<0.001	88.5
North America	5	6805	R, d‐l	PP = 6.30% (2.33–11.84)		<0.001	96.6
South America	3	1140	R, d‐l	PP = 3.97% (2.15–6.26)		0.17	44.3
Oceania	2	399	R, d‐l	PP = 12.53% (0.00–64.61)		<0.001	93.8
Study design^c^							
Prospective	8	15,136	R, d‐l	PP = 5.98% (2.60–10.57)	0.67^e^	<0.001	98.7
Retrospective	47	26,095	R, d‐l	PP = 6.71% (5.26–8.31)		<0.001	94.9
Time periods^c^							
Up to 1978	12	12,108	R, d‐l	PP = 5.35% (2.35–9.35)	0.75^e^	<0.001	97.8
From 1979 to 2007	13	4113	R, d‐l	PP = 7.06% (3.27–12.05)		<0.001	96.2
After 2007	30	25,010	R, d‐l	PP = 6.97% (5.50–8.59)		<0.001	94.2
Tobacco^d^							
Positive versus negative	12	10,410	F, m‐h	RR = 1.64 (1.25–2.15)	<0.001	<0.001	83.8
Alcohol^d^							
Drinkers versus non‐drinkers	8	1576	F, m‐h	RR = 1.26 (0.92–1.72)	0.14	0.16	33.1
Betel quid use^d^							
Chewers versus non‐chewers	1	2101	—	RR = 162 (10.05–2611.7)	<0.001	—	0.0
Sex^d^							
Female versus male	32	23,956	F, m‐h	RR = 1.39 (1.19–1.62)	<0.001	0.13	22.1
Age^d^							
Older (≥50 years) versus younger	6	1855	F, m‐h	RR = 1.57 (1.00–2.48)	0.051	0.28	20.1
Size (large vs. small)^d^					0.77		
All	6	978	F, m‐h	RR = 2.08 (1.45–2.96)	<0.001	0.20	31.8
200 mm^2^ versus <200	4	636	F, m‐h	RR = 2.17 (1.30–3.64)	0.003	0.20	35.2
400 mm^2^ versus <400	2	342	F, m‐h	RR = 1.96 (1.22–3.15)	0.005	0.10	62.3
Clinical type^b^							
NHL versus HL	18	3063	F, m‐h	RR = 4.23 (3.31–5.39)	<0.001	<0.001	59.3
Clinical type							
HL^b^	18	2004	R, d‐l	PP = 5.02 (1.87–9.22)		<0.001	90.0
NHL^b^	18	1059	R, d‐l	PP = 21.88 (16.44–27.81)		<0.001	73.6
Site‐distribution^c^							
Oral cavity (mixed)	52	41,049	R, d‐l	PP = 6.34 (4.93–7.89)	<0.001^e^	<0.001	96.8
Tongue and floor of mouth	3	182	R, d‐l	PP = 16.12 (10.92–22.03)		0.97	0.0
Anatomical site^d^							
Tongue versus others	21	10,442	F, m‐h	RR = 2.04 (1.72–2.41)	<0.001	<0.001	69.7
Tongue subsite^d^							
Border of tongue versus others	3	1770	F, m‐h	RR = 2.09 (1.48–2.95)	<0.001	0.001	85.7
Localization of lesions							
Tongue^b^	23	3331	R, d‐l	PP = 12.71 (8.09–18.07)		<0.001	91.9
Buccal mucosa^b^	22	9463	R, d‐l	PP = 3.95 (1.54–7.09)		<0.001	94.1
Floor of mouth^b^	19	431	R, d‐l	PP = 5.32 (2.81–8.34)		0.45	0.0
Palate^b^	19	842	R, d‐l	PP = 1.04 (0.00–3.65)		<0.001	63.3
Lips^b^	12	1351	R, d‐l	PP = 5.99 (0.12–16.49)		<0.001	88.6
Gingiva^b^	21	1648	R, d‐l	PP = 1.89 (0.48–3.89)		<0.001	65.9
Retromolar trigone^b^	1	46	‐	PP = 4.35 (1.20–14.53)		‐	0.0
Oral epithelial dysplasia^d^							
Presence versus absence	23	10,836	F, m‐h	RR = 2.75 (2.26–3.35)	<0.001	0.002	51.7
Dysplasia grade (binary)^d^							
High grade versus low grade	6	4075	F, m‐h	RR = 2.97 (2.25–3.91)	<0.001	0.98	0.0
Dysplasia							
No epithelial dysplasia^b^	22	5328	R, d‐l	PP = 2.38 (0.84–4.45)		<0.001	90.0
Mild dysplasia^b^	16	2130	R, d‐l	PP = 6.95 (2.79–12.30)		<0.001	83.8
Moderate dysplasia^b^	13	1213	R, d‐l	PP = 11.30 (5.13–19.03)		<0.001	83.0
Severe dysplasia^b^	12	398	R, d‐l	PP = 16.54 (4.31–32.61)		<0.001	80.1

Abbreviations: CI, confidence intervals; d‐l, DerSimonian and Laird method; ES, effect size estimation; F, fixed‐effects model; HL, homogeneous leukoplakias; m‐h, Mantel–Haenszel method; NHL, non‐homogeneous leukoplakias; PP, pooled proportion; R, random‐effects model; RR, relative risk; Stat., statistical.

^a^ Fifty two published studies, meta‐analyzed as 55 different units of analysis.

^b^ Proportion meta‐analysis (all studies).

^c^ Proportion meta‐analysis (Subgroups analysis).

^d^ Prognosis meta‐analysis.

^e^ Test for between‐subgroup differences.

**FIGURE 4:**
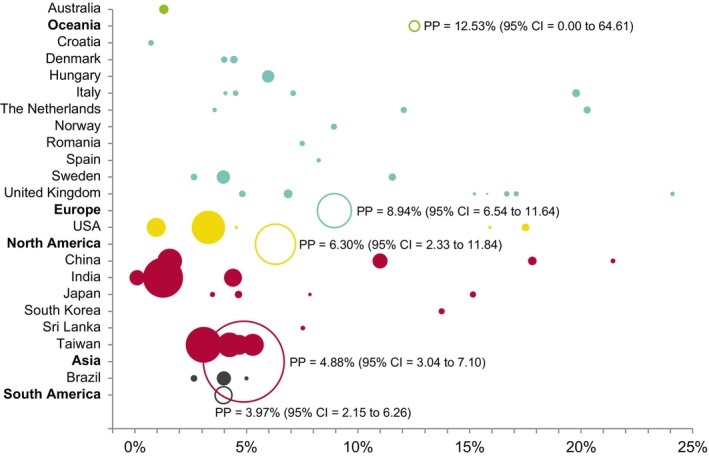
Bubble chart graphically representing the malignant transformation of oral leukoplakia according to countries and geographical regions (y‐axis). Filled bubbles correspond to the individual studies performed in each country. The order of bubbles represents the malignant transformation proportion of oral leukoplakia (x‐axis). Empty bubbles correspond to meta‐analyzed estimations by geographical region. The bubble diameter (z‐axis) is proportional to the sample size.

The malignant transformation of OL significantly varied among geographical regions (*p* < 0.001) (Figure 4), most studies were carried out in Europe (*n* = 25, PP = 8.94%, 95% CI = 6.54–11.64) and Asia (*n* = 20, PP = 4.88%, 95% CI = 3.04–7.10). On the other hand, clinic‐based studies (PP = 8.06, 95% CI = 6.23–10.08) showed a significantly higher malignant transformation proportion (*p* < 0.001), in comparison to population‐based studies (PP = 3.10%, 95% CI = 1.50–5.22). The results did not significantly vary in the stratified meta‐analyses by study design (*p* = 0.67).

Furthermore, the probability of oral cancer development in patients with OL was significantly higher among smokers (RR = 1.64, 95% CI = 1.25–2.15, *p* < 0.001) and females (RR = 1.39, 95% CI = 1.19–1.62, *p* < 0.001). The malignant transformation was also significantly higher in larger size OL (RR = 2.08, 1.45–2.96, *p* < 0.001), regardless of the cut‐off point applied (200 mm^2^: *p* = 0.003; 400 mm^2^: *p* = 0.005). According to clinical types, the non‐homogeneous leukoplakias harbored a higher malignant transformation (RR = 4.23, 95% CI = 3.31–5.39, *p* < 0.001; non‐homogeneous: PP = 21.88, 95% CI = 16.44–27.81). In relation to the anatomical site of OL, lingual location showed the highest malignancy proportion; tongue: PP = 12.71%, 95% CI = 8.09–18.07), followed by lips (PP = 5.99%, 95% CI = 0.12–16.49) and floor of mouth (PP = 5.32%, 95% CI = 2.81–8.34). Finally, the presence of epithelial dysplasia showed a significantly higher malignant transformation probability (RR = 2.75, 95% CI = 2.26–3.35, *p* < 0.001), with a malignant transformation proportion that progressively increases with the grade of epithelial dysplasia (high grade vs. low grade: no dysplasia: PP = 2.38%, 95% CI = 0.84–4.45; mild dysplasia: PP = 6.95%, 95% CI = 2.79–12.30; moderate dysplasia: PP = 11.30, 95% CI = 5.13–19.03; severe dysplasia: PP = 16.54%, 95% CI = 4.31–32.61; RR = 2.97, 95% CI = 2.25–3.91, *p* < 0.001).

### 3.5 Quantitative evaluation (secondary analyses)

In the sensitivity analysis, the sequential repetition of the meta‐analysis by performing the so‐called “leave‐one‐out” method (Figure S27 in supplementary information) did not influence on the overall resultant pooled proportion. Therefore, the reported malignant transformation proportion of OL is stable.

In the small‐study effects analysis, the visual inspection of the asymmetry of the funnel plot (Figure S28 in supplementary information) and the statistical test performed for the same purpose (*p*_Egger_ <0.001) confirmed the significant presence of small‐study effects. Therefore, it is not possible to rule out publication bias.

## 4 DISCUSSION

Our systematic review and meta‐analysis of malignant transformation of oral leukoplakia, including 55 primary‐level studies and 41,231 patients, yields a malignant transformation probability for OL of 6.64% (95% CI = 5.21–8.21). This malignancy rate is lower than what was earlier reported in published meta‐analytic studies on the subject. The observed differences could arise from widening the limits of the literature search without restriction by date of publication or language; in addition, the eligibility criteria used here have allowed us to eliminate interventional studies, in which therapeutic procedures were applied that could affect the rate of malignization.

To further examine the potential influence of the study period on MT rates, we stratified the analysis into three groups; as studies conducted prior to the first publication by WHO in 1978 that provided a definition of OL, then from that time to the WHO consensus report in 2007 and the third period from 2007 onwards. We found that although the malignancy rate of the first period was slightly lower (5.35%) compared to the rates of the other two periods (7.06% and 6.97%, respectively), the differences among them were not significant (*p* = 0.75). This slightly lower rate found in the first period may have been related to lack of precise definitions used to classify oral leukoplakia during this early period prior to the first WHO report on this subject; these primary studies may have included other white lesions, that is, frictional keratoses when diagnosing oral leukoplakia. On the other hand, a recent meta‐analysis (Aguirre‐Urizar et al., 2021) that included studies over the 5 years preceding its publication in 2021, has reported a higher malignancy rate of OL (9.8%). Whether this actually represents a trend toward an increased probability of malignant transformation of OL is something to be clarified by future primary‐level studies on the subject.

The primary‐level studies included in our meta‐analysis are population‐based (12 papers, 12,514 patients), in which the authors have searched for cases of OL in the community – for example by house‐to‐house visiting or enrolling textile industry workers (Chaturvedi et al., 2020; Gupta et al., 1980; Silverman et al., 1976)‐ and studies in specialized clinics – essentially university clinics of Oral Medicine and Stomatology – where patients with OL who have been specifically referred for diagnosis and management had attended these units (43 papers, 28,717 patients). In both types of studies, patients underwent follow‐up of their lesion. The malignancy rate derived from our meta‐analysis in studies conducted in specialized clinics (8.06%, 95% CI = 6.23–10.08) was significantly higher (*p* < 0.001) than that obtained from population‐based studies (3.10%, 95% CI = 1.50–5.22). In our opinion, this difference derives from the fact that patients referred to specialized clinics would presumably present lesions with more severity which, as we will see below, could detrimentally affect the probability of malignant transformation. Furthermore, implicit in this result is also the fact that a considerable number of less severe OL are likely to be found in population‐based studies. Our meta‐analysis also demonstrates significant geographical differences (*p* = 0.03) in the malignancy of OL, with Europe being the area of the world with the highest malignancy rate (8.94%, 95% CI = 6.54–11.64) and Asia the lowest (4.88%, 95% CI = 3.04–7.10). For the remaining geographical areas of the world (North America, South America and Oceania), the evidence is less robust as there are few primary‐level studies conducted on this topic. In our study, OL in tobacco users (in any form, although inhaled tobacco use was the most common) had a significantly higher probability of developing cancer (*p* < 0.001) compared to OL in non‐users (RR = 1.64, 95% CI = 1.25–2.15). This is relevant not only because this observation differs from the commonly cited legend that idiopathic OL has a higher malignancy ratio, but also because it points out that patients with OL who use tobacco should necessarily quit as a preventive measure to reduce the probability of a future malignancy. The fact that OL in smokers have a higher malignancy ratio could be justified in our opinion because of the sustained aggression of smoking hypothetically increases the probability of acquiring new additive molecular oncogenic events on already genetically altered cell clones.

In relation to demographic factors, our study indicates that OL could significantly have more malignant potential in women (RR = 1.39, 95% CI = 1.19–1.62; *p* < 0–001), with a trend toward significance in patients over 50 years old (RR = 1.57, 95% CI = 1.00–2.48; *p* = 0.051), which would presumably reach a discriminative significance level if the number of studies analyzing this aspect were increased.

According to clinical factors associated with malignancy of a lesion, we found that larger OL have a higher probability of developing cancer than smaller ones (RR = 2.08, 95% CI = 1.45–2.96; *p* < 0.001), regardless of the cut‐off point used by the authors for comparison: 200 mm^2^ or 400 mm^2^. Although there is no evidence‐based justification for this result, logic dictates that the greater the surface area of diseased tissue, the greater the likelihood that any cell clone will achieve the molecular alterations necessary for transformation. Our meta‐analysis also demonstrates that non‐homogeneous leukoplakias have a much higher probability of malignancy than homogeneous leukoplakia (RR = 4.23, 95% CI = 3.31–5.39; *p* < 0.001), with 21.88% of non‐homogeneous clinical types developing cancer versus 5.02% of homogeneous leukoplakia. This result directs clinicians to implement a diligent approach in the management of these mixed red and white or speckled lesions. The rationale for this finding is probably due to the fact that non‐homogeneous leukoplakia, especially erythroleukoplakia, harbor red areas that in some cases could correspond to severe dysplasia (González‐Moles et al., 2022; González‐Ruiz et al., 2023). Moreover, our results indicate that the tongue constitutes the site of higher probability of malignancy (RR = 2.04, 95% CI = 1.72–2.41; *p* < 0.001), where 12.71% of OL become malignant, with the lateral border of the tongue being specifically the location of highest malignant transformation (RR = 2.09, 95% CI = 1.48–2.95; *p* < 0.001). Other areas notably predisposed are the floor of the mouth (5.32%) and the lips (5.99%).

Finally, the morphological alterations found by histopathological analysis of the affected tissue also contribute as determinant risk factors for malignancy. The presence of epithelial dysplasia significantly increases the malignant transformation, which almost triples (RR = 2.75, 95% CI = 2.26–3.35; *p* < 0.001). Furthermore, although high‐grade dysplasia is, as expected, associated with the highest malignant transformation (RR = 2.97, 95% CI = 2.25–3.91; *p* < 0.001), our study interestingly reflects that any grade of dysplasia is associated with a significant probability of malignancy (mild dysplasia 6.95%, moderate dysplasia 11.30%, severe dysplasia 16.54%); this meta‐analysis dismantles the classical belief that OL without dysplasia (2.38% of malignant cases) and those with mild dysplasia are of little concern. Clinicians should therefore act proactively on all OL, with biopsy being imperative in all cases but irrespective of the grade of dysplasia found in the biopsy.

As a potential limitation that should be discussed, this systematic review and meta‐analysis does not give the dimension of the true risk for oral cancer at individual and population levels, but suggests that a given proportion of leukoplakia cases (6.6%) will transform into cancer, somewhere in time. Currently, a limited number of primary‐level studies have reported accurately the malignant transformation risk of oral leukoplakias, defined as the probability of an event during a specified period of time, preferably in the form of annual incidence rates. Future studies are needed in order to provide this valuable specific information for clinicians and public health officers. Furthermore, most studies report aggregated data for the analyzed variables of interest, making it impossible to perform subgroup analyses among all the investigated risk factors. In addition, not all the included studies reported detailed data for the whole demographic and clinicopathological characteristics of patients with OL (e.g., age). However, these criticisms are truly inherent limitations of primary‐level studies, which should be more rigorous in their reporting. Thus, the present systematic review and meta‐analysis recommends that future studies should diligently report individual participant data, in order to comprehensively analyze the totality of characteristics influencing the malignant transformation of OL. It would be desirable to develop a minimal data set for reporting outcomes for use in future primary‐level studies.

In conclusion, oral leukoplakia is an OPMD which presents an important malignant transformation probability that is especially increased in large OL, non‐homogeneous lesions, located on the lateral border of the tongue, presenting epithelial dysplasia, and in smokers.

## AUTHOR CONTRIBUTIONS

**Liliana Aparecida Pimenta‐Barros:** Conceptualization; investigation; methodology; validation; writing – review and editing; visualization; software; formal analysis; data curation; resources; supervision. **Pablo Ramos‐García:** Conceptualization; investigation; writing – original draft; methodology; validation; visualization; writing – review and editing; software; formal analysis; data curation; supervision; resources. **Miguel Ángel González‐Moles:** Conceptualization; investigation; writing – original draft; methodology; validation; visualization; writing – review and editing; software; formal analysis; data curation; resources; supervision. **José Manuel Aguirre‐Urizar:** Conceptualization; data curation; investigation; validation; supervision; visualization; resources; writing – review and editing. **Saman Warnakulasuriya:** Conceptualization; investigation; validation; visualization; writing – review and editing; data curation; supervision; resources.

## CONFLICT OF INTEREST STATEMENT

All authors have no conflicts of interest to disclose.

## Supporting information


Data S1.

## Data Availability

Data are contained within the article or supplementary material.
