# Supplementary material for: Malignant transformation of oral leukoplakia: Systematic review and comprehensive meta‐analysis
Source: Oral Dis. 2024 Sep 24;31(1):69–80. doi: 10.1111/odi.15140 (PMC11808172; doi:10.1111/odi.15140)
Supplement: Supplementary file 1 — Data S1. [file ODI-31-69-s001.pdf]

Appendix to the manuscript

Malignant transformation of oral leukoplakia: a systematic review and comprehensive meta-analysis

Table of contents

1. Search strategy..3

2. Descriptive characteristics of the study sample..4

3. Meta-analyses on malignant transformation of oral leukoplakia 9

4. Sensitivity analysis. 36

5. Analysis of small-study effects..37

6. List of included studies..38

7. List of excluded studies with reasons...44

1. Search strategy

Table S1. Search strategy for each database, number of results, and execution date.

Database	Query/Search Strategy	Results/ Items found	Search time limits
MEDLINE	("Leukoplakia, Oral"[Mesh] OR ("leukoplakia"[All Fields] AND ("Mouth"[Mesh] OR "oral"[All Fields]))) AND (malign* OR premalign* OR precancer* OR "potentially malignant disorder" OR "cancer"[All Fields] OR "Carcinoma, Squamous Cell"[MeSH] OR "squamous cell carcinoma"[All Fields] OR "oscc"[All Fields] OR "transformation" [All Fields] OR "risk"[All Fields] OR "progression"[All Fields])	4,158	June, 2024
Embase	(('leukoplakia'/exp OR 'leukoplakia') AND ('mouth'/exp OR 'oral')) AND ('malign*' OR 'premalign*' OR 'potentially malignant disorder' OR 'precancer'/exp OR 'precancer' OR 'cancer'/exp OR 'cancer' OR 'squamous cell carcinoma'/exp OR 'squamous cell carcinoma' OR 'oscc' OR 'transformation'/exp OR 'transformation' OR 'risk'/exp OR 'risk' OR 'progression')	7,594	June, 2024
Web of Science	TS=(leukoplakia AND (oral OR mouth)) AND TS=(malign* or premalign* or potentially malignant disorder or precancer or cancer or squamous cell carcinoma or oscc or transformation or risk or progression)	3,499	June, 2024
Scopus	TITLE-ABS-KEY (("leukoplakia" AND ("oral" OR "mouth")) AND ("malign*" OR "premalign*" OR "potentially malignant disorder" OR "precancer" OR "cancer" OR "squamous cell carcinoma" OR "oscc" OR "transformation" OR "risk" OR "progression"))	5,693	June, 2024
Total			20,944

Table S2. Descriptive characteristics of the study sample

Author (year); Country	Study design (recruitment period)	Follow up, months mean±SD (range)	Sample size, n	sex, n (%)	age, y mean±SD	Anatomical sites, n	Tobacco	Alcohol	Betel quid	Clinical type, n	Epithelial dysplasia, n (grading)	Malignant transformation	
												Ratio, n (%)	mean time, months
Sturgis and Lund, (1934); USA	Retrospective cohort (1918-1926)	NR±NR<br>(0- >60)	n=44	NR	NR	NR	NR	NR	NR	NR	NR	2<br>(4.55%)	NR
Leonardelli and Talamazzi, (1950); Italy	Retrospective cohort (1928-1948)	NR±NR<br>(0- 240)	n=268	M=218 (81.34%)<br>F=50 (18.66%)	<50y=79<br>(NR±NR)<br>>50y=<br>189(NR±NR)	Tongue=96<br>Gingiva=25<br>Buccal mucosa=75<br>Palate=25<br>Lip=47	NR	NR	NR	NR	NR	53<br>(19.78%)	NR<br>(3-180)
Mela and Mongini, (1966); Italy	Retrospective cohort (1934-1962)	NR±NR<br>(36-132)	n=141	M=117 (82.9%)<br>F=24 (17.1%)	NR	NR	NR	NR	NR	NR	NR	10<br>(7.09%)	NR
Einhorn and Wersäll (1967); Sweden	Retrospective cohort (1920-1960)	140.4±NR<br>(12-528)	n=782	M= 522<br>(66.75%)<br>F=260<br>(33.25%)	20-49y=<br>274<br>50-69y=<br>399<br>70-89y=<br>109	NR	Smokers =<br>649(83%)<br>Non-smokers<br>133(17%)	NR	NR	NR	NR	31<br>(3.96%)	NR
Pindborg et al. (1968); Denmark	Prospective cohort (1955-1964)	median=44,4<br>(0-120)	n=248	M= 133<br>(53.63%)<br>F=81<br>(32.66%)	NR	NR	NR	NR	NR	NR	NR	11<br>(4.44%)	NR
Kramer and Path, (1969); UK	Retrospective cohort	NR±NR<br>(1-192)	n=187	NR	NR	NR	NR	NR	NR	NR	NR	9<br>(4.8%)	NR
Gangadharan and Paymaster (1971); India	Prospective cohort (1941-1970)	NR±NR<br>(3 to 276)	n=1411	M=1147(80.66%)<br>F=264(18.56%)	45.84±12.12	Buccal mucosa=889<br>Tongue= 301<br>Lips=40<br>Palate=29<br>FOM=10 Gingiva=11<br>Multiple sites= 131	NR	NR	NR	NR	NR	62<br>(4,39%)	8.5<br>(3-276)
Waldron and Shafer, (1975); USA	Retrospective cohort (1960-1973)	NR±NR<br>(12-120)	n=1574	M= 795<br>(50.5%)<br>F=779<br>(49.5%)	NR	NR	NR	NR	NR	NR	No OED= 1134<br>OED=440	15<br>(0.95%)	NR
Silverman et al. (1976); India	Prospective cohort (1967-1971)	NR±NR<br>(0-24)	n=6718	M=6382(95%)<br>F=336(5%)	NR	Buccal mucosa=5166<br>Labial mucosa=981<br>Tongue= 229<br>Gingiva=181<br>Palate=141<br>Floor Mouth=20	Smokers =<br>2768(41,2%)<br>Non-smokers<br>114(1,7%)<br>Smokers+tobacco<br>chewing=302(4,5%<br>)	NR	Smoking +<br>pan/supari<br>chewing=22<br>64(33,7%)	NR	No OED=6678<br>OED =35	6<br>(0,09%)	NR
Bánóczy, (1977); Hungary	Prospective cohort (1946-1976)	117.6±NR<br>(12-360)	n=670	M=510 (76.12%)<br>F=160 (23.88%)	21-30y=<br>15<br>31-40y=<br>67<br>41-50y=<br>132<br>51-60y=<br>201<br>61-70y=<br>182<br>>71=<br>73	Gingiva=45<br>Buccal mucosa=421<br>Tongue=55<br>Hard Palate=57<br>Soft Palate=9<br>FOM=38<br>Lip=45	Smokers=583<br>Non-smokers=87	Yes=101<br>No=569	NR	HL= 371<br>NHL=299	No OED=22<br>OED=98<br>NR=550	40<br>(5.97%)	117.6<br>(12-360)
Kramer, El-Labban and Lee, (1978); UK	Retrospective cohort (1955-1977)	50.4±NR<br>(12-228)	n=46	NR	NR	Sublingual region=46	NR	NR	NR	HL=42<br>NHL=3<br>NR=1	NR	7<br>(15.21%)	NR
Pogrel, (1979); UK	Retrospective Serie cases	NR±NR<br>(60-180)	n=19	M=7 (63.15%)<br>F=12 (36.85%)	48-73<br>(NR±NR)	FOM=19	Smokers=8<br>Non-smokers=11	NR	NR	NR	NR	3<br>(15.78%)	NR
Gupta et al., (1980); India	Prospective Cohort (1969-1977)	84±NR<br>(12-120)	n=2460	NR	NR	NR	NR	NR	NR	NR	NR	13<br>(0,53%)	NR

Roch-Berry, (1981);<br>UK	Retrospective<br>(1932-1972)	NR±NR	n=117	M= 68<br>(58.12%)<br>F=49<br>(41.88%)	NR	Tongue=117	NR	NR	NR	NR	NR	20<br>(17.10%)	NR
Silverman et al.(1984);<br>USA	Retrospective<br>cohort	86,4±NR<br>(6-468)	n=257	M=125(48.64%)<br>F=132(51.36%)	54y±NR	Buccal mucosa=118<br>Gingiva=103<br>Palate=69<br>Tongue= 67<br>FOM=56 Lip=28	Smokers =<br>183(71.20%)<br>Non-smokers<br>74(28.80%)	NR	NR	HL= 107<br>NHL=148<br>Undefined=2	NR	45<br>(17,50%)	97,2<br>(12-468)
Lind, (1987); Norway	Retrospective<br>(1970-1980)<br>Prospective<br>(1980-1986)	116.6±NR<br>(3-192)	n=157	M= 102<br>(65%)<br>F=55<br>(35%)	57.7y=<br>(13.8±NR)	NR	Smokers =<br>46(29.3%)<br>Non-smokers<br>111(70.70%)	NR	NR	HL= 35<br>NHL=96<br>NR=24	No OED =105<br>OED =38<br><br>(Mild:15<br>Moderate:19<br>Severe:4)	14<br>(8.92%)	66<br>(2-189)
Hogewind, van der Kwast, van der Wall, (1989); The Netherlands	Retrospective<br>(1969-1984)	30±NR<br>(12-96)	n=84	M=50 (59.5%)<br>F=34 (40.5%)	16-30y=<br>6<br>31-45y=<br>17<br>46-60y=<br>24<br>>60y=<br>37	Gingiva=7<br>Buccal mucosa=18<br>Tongue=10<br>Palate=4<br>FOM=3<br>Lip=3<br>Pharynx=2<br>Combinations=35<br>NR=2	Smokers =<br>75(82.28%)<br>Non-smokers=<br>9(10.72%)	NR	NR	HL=11<br>NHL=22<br>NR=51	No OED=22<br>OED =10<br>NR=14<br><br>(Mild: 2<br>Moderate:3<br>Severe:5)	3<br>(3.57%)	50<br>(30-84)
Lumerman et al., (1995);<br>USA	Retrospective<br>(1974-1982)	18.4±NR<br>(6-113)	n=44	M=20 (45.45%)<br>F=24 (54.55%)	61.3±NR<br>(28-89)	Gingiva=5<br>Buccal mucosa=6<br>Tongue=17<br>Palate=3<br>FOM=11<br>Lip=2	NR	NR	NR	HL=25<br>NHL=14<br>NR=5	No OED= 0<br>OED=44<br><br>(Mild:19<br>Moderate:18<br>Severe:7)	7<br>(15.9%)	33.6<br>(7-78)
Schepman et al., (1998);<br>The Netherlands	Retrospective<br>(1973-1997)	median=29<br>(6-209)	n=166	M=76(46.78%)<br>F=90(54.22%)	57±NR	Gingiva=20<br>Buccal mucosa=26<br>Tongue=54<br>Palate=3<br>FOM=32<br>Lip=6<br>Multiple sites=25	Smokers=93<br>Non-smokers=58<br>NR=15	Yes=85<br>No=42<br>Unknow+30<br><2U/day	NR	HL=99<br>NHL=57<br>NR=10	No OED=47<br>OED=62<br><br>(Mild:20<br>Moderate:24<br>Severe:18)<br><br>Not specified=13<br>No biopsy=44	20<br>(12%)	median=32<br>(6-201)
Saito et al., (2001);<br>Japan	Retrospective<br>(1976-1997)	48±NR<br>(7-192)	n=51	NR	54±NR	Gingiva=15<br>Buccal mucosa=13<br>Tongue=12<br>Palate=7<br>FOM=4	NR	NR	NR	NR	No OED=17<br>OED=34<br><br>(Mild:17<br>Moderate:16<br>Severe:1)	4<br>(7.84%)	60<br>(36-72)
Napier et al., (2003);<br>Northern Ireland, UK	Retrospective<br>(1975-1994)	88,7±NR<br>(22-174)	n=50	M=18 (36%)<br>F=32 (64%)	58±NR<br><50y=14<br>>50y=36	Single lesion=23<br>Multiple lesions=27	Smokers=40<br>Non-smokers=10	NR	NR	HL= 28<br>NHL=22	NR	17<br>(34%)	71<br>(22-156)
Holmstrup et al., (2006);<br>Netherlands	Retrospective<br>(1977-1997)	66±NR<br>(13,2-242,4)	n=175	M=75 (42.86%)<br>F=100 (57.14%)	60.6±NR	Gingiva=21<br>Buccal mucosa=80<br>FOM=40<br>Ventral tongue=10<br>Lateral border tongue=2<br>Dorsum tongue=2<br>Palate=8<br>Lip=12	Smokers=142<br>Non-smokers=33	NR	NR	HL=149<br>NHL=26	No OED =89<br>OED =21<br><br>(Mild:14<br>Moderate:4<br>Severe:3)<br><br>No biopsy=65	7<br>(4%)	79.2<br>(12-206.4)
Hsue et al., (2007);<br>Taiwan	Retrospective<br>(1991-2001)	42.2±NR	n=552	NR	47.5±NR	NR	Smokers=552<br>Non-smokers=0	Yes=552<br>No=O	NR	NR	No OED= 423<br>OED=129	21<br>(3.80%)	34.75**
Arduino et al., (2009);<br>Italy	Retrospective cohort<br>(1991-2007)	median=54<br>(12-192)	n=74	NR	63.8±10.72	NR	NR	NR	NR	NR	No OED= 0<br>OED=74<br><br>(Mild:55<br>Moderate:13<br>Severe:6)	3<br>(4.05%)	30.3<br>(24-35)
Warnakulasuriya et al., (2011); UK	Retrospective<br>(1990-2005)	108.5±NR	n=335	NR	NR	NR	NR	NR	NR	NR	NR	23<br>(6.87%)	NR

Brzak et al., (2012); Croatia	Retrospective<br>(1998-2007)	NR±NR<br>(1-120)	n=139	M=60 (43%)<br>F=79(57%)	49±17<br>(22-70)<br><br>median=41.1	NR	Smokers=65<br>Non-smokers=53<br>NR=21	NR	NR	NR	No OED= 121<br>OED=18	1<br>(0,72%)	48
Liu et al., (2012); China	Retrospective cohort<br>(1990-2010)	61.2±NR<br>(12-240)	n=320	M=145 (45.31%)<br>F=175 (54.69%)	54.1±11.6<br>(21-83)	Buccal mucosa=93<br>Ventral/lateral tongue=121<br>Dorsal tongue=62<br>Gingiva=26<br>Palate=12<br>Others sites=6	Smokers+ Ex=86<br>Non-smokers=194<br>NR=40	Yes=84<br>No=196<br>NR=40	NR	HL=301<br>NHL=19	Low=229<br>High=91	57<br>(17.8%)	54
Ho et al., (2012); UK	Retrospective<br>(1984-1999)<br>Prospective<br>(1999-2011)	median= 48	n=83	M= 45<br>(54.22%)<br>F=38<br>(45.78%)	58.3±NR<br>(37.5-85.2)	FOM=39<br>Buccal mucosa=15<br>Lateral tongue=14<br>Ventral tongue=4<br>Dorsal tongue=1<br>Gingiva=4<br>Palate=6	Smokers=66<br>Non-smokers=17	Yes=44<br>No=42<br>NR=3	NR	HL=65<br>NHL=18	No OED= 0<br>OED=83<br><br>(Mild:40<br>Moderate:28<br>Severe:15)	20<br>(24.1%)	94.4<br>(13.6-257.1)
Wang et al., (2014); Taiwan	Retrospective cohort<br>(2001-2010)	NR±NR<br>(3-120)	n=2641	M=2250 (85.2%)<br>F=391 (14.8%)	49.77±13.38*	Gingiva=396<br>Buccal mucosa=1483<br>Tongue=415<br>Palate=122<br>FOM=46<br>Lip=179	Smokers=2281<br>Non-smokers=360	Yes=1928<br>No=713	Yes=2341<br>No=300	NR	No OED= 1684<br>OED=957	112<br>(4.24%)	32.21**
Kuribayashi et al., (2015); Japan	Retrospective<br>(2001-2010)	median=41.1<br>(12-126)	n=237	M=131 (55.28%)<br>F=106 (44.72%)	<60=99(NR±NR)<br>>=60=138(NR±NR)	Gingiva=72<br>Buccal mucosa=57<br>Tongue=64<br>Palate=35<br>FOM=2<br>Lip=7	Smokers=76<br>Ex=28<br>Non-smokers=41<br>NR=73	NR	NR	HL=200<br>NHL=37	No OED=124<br>OED=113<br><br>(Mild:72<br>Moderate:40<br>Severe:1)	11<br>(4.65%)	NR
Lima et al., (2016); Brazil	Retrospective<br>(2005-2011)	NR<br>(0-48)	n=73	M=36 (49.32%)<br>F=36<br>(49.32%)<br>NR=1(1.36%)	NR	Gingiva=14<br>Buccal mucosa=18<br>Tongue=22<br>Palate=5<br>FOM=11<br>NR=3	Smokers=39<br>Non-smokers=34	NR	NR	NR	No OED=44<br>OED=29	6<br>(8.22%)	7.25**
Watabe et al., (2016); Japan	Retrospective<br>(2000=2011)	NR±NR<br>(2-120)	n=115	M=65 (56.5%)<br>F=50 (43.5%)	62.8±10.2	Gingiva=43<br>Buccal mucosa=12<br>Tongue=42<br>Palate=13<br>FOM=4<br>NR=1	Smokers + Ex=26<br>Non-smokers=52<br>NR=37	Yes=44<br>No=33<br>NR=38	NR	HL=69<br>NHL=12<br>NR=34	No OED= 0<br>OED=115<br><br>(Mild:89<br>Moderate:26<br>Severe:0)	4<br>(3,48%)	40.25<br>(13-69)
Tsai et al., (2017); Taiwan	Retrospective cohort<br>(2000-2014)	NR±NR<br>(1-168)	n=2101	M=2101(100%)	46.97±12.99	NR	Smokers=1329<br>Non-smokers=772	NR	Yes=985<br>No=1116	NR	NR	111<br>(5.28%)	87.48±54
Zhang et al., (2017); South Korea	Retrospective<br>(1994-2009)	median=135.6<br>(55.2-278.4)	n=160	M=100 (62.5%)<br>F=60 (37.5%)	51.9±NR<br><br>median=54	Gingiva=72<br>Buccal mucosa=44<br>Tongue=44	NR	NR	NR	NR	No OED= 82<br>OED=78<br><br>(Low=54<br>High=24)	22<br>(13.8%)	39.6<br>(12-124.8)
Chuang et al., (2018); Taiwan	Prospective<br>(2004-2012)	68.4±NR<br>(1-96)	n=5616	M=5616(100%)	47.5**±NR	NR	NR	NR	NR	HL=5616<br>NHL=204	NR	173<br>(3.08%)	NR
Gandara-Vila et al., (2018); Spain	Retrospective<br>(1995-2010)	49.56±NR<br>(95%CI:<br>40.32-58.68)	n=85	M=45 (52.94%)<br>F=40 (47.06%)	58.68±12.88<br>(22 to 85)	Gingiva=19<br>Buccal mucosa=19<br>Tongue=32<br>Palate=5<br>FOM=7<br>Lip=3	Smoker + Ex=38<br>Non-smokers=47	Yes=28<br>No=50<br>NR=7	NR	HL=36<br>NHL=49	No OED=59 OED=26<br><br>(Mild:19<br>Moderate:4<br>Severe:3)	7<br>(8.24%)	66.96<br>(95%CI: 13.8-120.24)
Wang et al., (2018); Taiwan	Retrospective cohort<br>(2001-2013)	55.2±39.6<br>(12-156)	n=1688	NR	NR	NR	NR	NR	NR	NR	NR	79<br>(4.68%)	21.6
Wu et al., (2018); China	Retrospective<br>(1996-2015)	median=95<br>(20-240)	n=98	M=45 (45.92%)<br>F=52 (54.08%)	median=49<br>(18-71)<br>median=56<br>(24-76)	Labial and buccal mucosa, gingiva, palate=50<br>Tongue and FOM=39	Smokers=23<br>Non-smokers=75	Yes=11<br>No=87	NR	NR	Low-grade=76<br>High-grade=22	21<br>(21.42%)	median=57<br>(13-173)
Chaturvedi et al., (2019); USA	Retrospective cohort<br>(1996-2012)	55.44±NR<br>(0-196)	n=4886	M=2784 (57%)<br>F=2102 (43%)	57.8±NR	NR	Smokers=2078<br>Non-smokers=2808	Yes=253<br>No=4633	NR	NR	NR	161<br>(3,29%)	NR

Farah et al., (2019);<br>Australia	Retrospective<br>(over a period of at least 10 year)	119.5±NR<br>(16-220)	n=13	M=6 (46.15%)<br>F=7 (53.85%)	58.3±NR	Buccal mucosa=3<br>Tongue=7<br>FOM=1<br>Gingiva=2	Smokers=9<br>Non-smokers=2	NR	NR	HL=3<br>NHL=10	No OED=3 OED=10<br><br>(Mild:7<br>Moderate:1<br>Severe:2)	5<br>(38.46%)	46.2
Shearston et al., (2019);<br>Australia	Retrospective<br>cohort<br>(2006-2014)	NR±NR<br>(18-168)	n=386	M=205<br>(53%)<br>F=181<br>(47%)	56.6±13.4	NR	NR	NR	NR	NR	NR	5<br>(1.3%)	58.8
Wu et al., (2019);<br>China	Retrospective cohort<br>(2000-2015)	65.8±NR<br>(6-180)	n=2628	M=1369 (52.1%)<br>F=1259 (47.9%)	NR	Buccal mucosa=739<br>Ventral Tongue=673<br>Lateral Tongue=476<br>Dorsal tongue=224<br>Gingiva=206<br>Palate=132<br>Others sites=178	NR	NR	NR	NR	No OED= 251<br>OED=2377<br><br>(Mild:1419<br>Moderate:733<br>Severe: 225)	41<br>(1.56%)	26.7
Jayasooriya et al.,<br>(2020); Sri Lanka	Retrospective<br>(2009-2013)	30±NR<br>(10-72)	n=93	M=73 (78.5%)<br>F=20 (21.5%)	<50y=<br>23(NR±NR)<br>>=50y=<br>70(NR±NR)	Buccal mucosa=66<br>Tongue or FOM=22<br>Other=5	NR	NR	NR	HL=58<br>NHL=38	No OED=20 OED=73<br><br>(Mild:34<br>Moderate:20<br>Severe:19)	7<br>(7.53%)	27.3**
Sakata et al., (2020);<br>Japan	Retrospective<br>(2000-2013)	36.35**±NR<br>(0-156)	n=165	M=89 (53.9%)<br>F=76 (46.1%)	median=63<br>(29-87)<br><br>median=64<br>(19-87)	Tongue=62<br>Gingiva=68<br>Buccal mucosa=21<br>Others=14	NR	NR	NR	NR	No OED= 116<br>OED=49<br><br>(Mild:36<br>Moderate/Severe:13)	25<br>(15,15%)	NR
Sundberg et al., (2020);<br>Sweden, Brazil and Romania***Romanian cohort was updated and meta-analyzed from Tovaru et al 2022	Retrospective<br>Multi-centre study<br>(1992-2002)<br>(2011-2017)	Sweden<br>NR±NR<br>(1-120)<br><br>Brazil<br>NR±NR<br>(1-72)	n=378<br><br>Sweden=189<br>Brazil=189	M=196 (51.85%)<br>F=182 (48.15%)	58.5.**±NR	FOM=49<br>Gingiva=100<br>Buccal mucosa=82<br>Tongue=121<br>Palate=26	NR	NR	NR	NR	No OED= 201<br>OED=177	Sweden: 5<br>(2.65%)<br><br>Brazil: 5<br>(2.65%)	Sweden:<br>34.2<br><br>Brazil:<br>25.4
Cai et al., (2021); China	Retrospective cohort<br>(2010-2017)	74±NR<br>(12-120)	n=983	NR	NR	NR	NR	NR	NR	NR	NR	108<br>(10.99%)	NR
Jäwert et al., (2021);<br>Sweden	Retrospective cohort<br>(2003-2013)	median=108<br>(3-234)	n=234	M=122 (52.14%)<br>F=112 (47.86%)	<60y=<br>102(NR±NR)<br>>=60y=<br>132(NR±NR)	Tongue=65<br>Gingiva=83<br>Buccal mucosa=33<br>Palate=15<br>FOM=24<br>Lip=14	Smokers=82<br>Non-smokers=131	NR	NR	HL=160<br>NHL=74	No OED=204 OED=30	27<br>(11.54%)	49
Tovaru et al., (2022);<br>Romania	Retrospective cohort<br>(1996-2014)	NR±NR<br>(12-192)	n=120	M=52 (43.4%)<br>F=68 (56.7%)	55.85±NR	Buccal mucosa=14<br>Tongue=10<br>Gingiva=29<br>FOM=5<br>Other=76	Smokers=71<br>Ex=15<br>Non-smokers=34	NR	NR	HL=72<br>NHL=48	No OED=75 OED=45<br><br>(Mild:34<br>Moderate:9<br>Severe:2)	9<br>(7.5%)	75<br>(12-180)
Sathasivam et al.,<br>(2022); UK	Retrospective cohort<br>(2000-2015)	median=62<br>(7-190)	n=126	NR	58.59±13.22	NR	NR	NR	NR	HL=110<br>NHL=16	NR	21<br>(16.7%)	26
Pentenero et al., (2023);<br>Italy	Prospective<br>(2008-2014)	93.6±36<br>(6-153.6)	n=133	M=67 (50.4%)<br>F=66 (49.6%)	60.08±13.98	Gingiva=25<br>Buccal mucosa=46<br>Tongue=28<br>Palate=19<br>FOM=13<br>Lip=2	Smokers=92<br>Non-smokers=41	No regular=81<br>Regular=52	NR	HL=109<br>NHL=24	No OED=112 OED=21<br><br>(Mild:18<br>Moderate:1<br>Severe:2)	6<br>(4.52%)	39.67
Evreb et al., (2023); The Netherlands	Retrospective cohort<br>(1997-2021)	median=64<br>(12-300)	n=222	M=75 (33.78%)<br>F=147 (66.21%)	60±13.1	Gingiva=44<br>Buccal mucosa=20<br>Tongue=72<br>Palate=16<br>FOM=35<br>Lip=3<br>Multiple site=32	Smokers=98<br>Non-smokers=101<br>NR=23	Yes=111<br>No=43<br>NR=68	NR	HL=123<br>NHL=99	No OED=142<br>OED=78<br><br>(Mild:36<br>Moderate:23<br>Severe:19)	45<br>(20.27%)	59<br>(14-244)

Sperandio et al., (2023); Brazil	Retrospective cohort (2005-2021)	NR±NR (6-192)	n=878	M=399 (45.44%) F=442 (50.34%) NR=37(4.21%)	<50y=231 >50y=603 NR=44	Gingiva=182 Buccal mucosa=209 Tongue=167 Palate=114 FOM=53 Retromolar=46 NR=107	NR	NR	NR	NR	No OED=262 OED=616 (Mild:259 Moderate:265 Severe:92)	5 (3.99%)	NR

3. Meta-analyses on malignant transformation of oral leukoplakia

3.1 Overall pooled proportion

Figure S1. Forest plot graphically representing the meta-analysis of the malignant transformation of oral leukoplakia. Pooled proportions (expressed as percentage) and 95% confidence intervals (CI) were used as effect size metric.

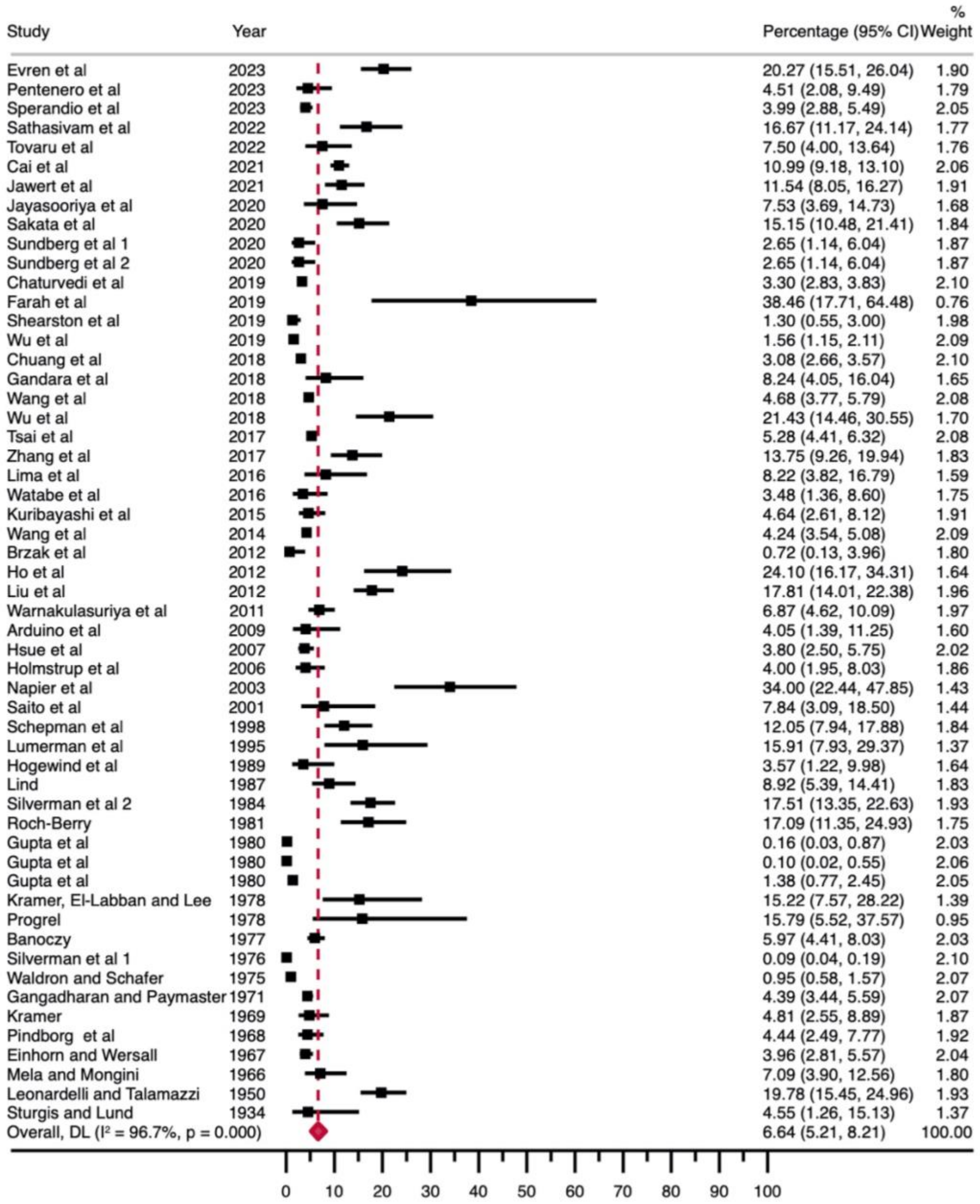

3.2 Subgroup meta-analysis stratified by geographical region

Figure S2. Forest plot graphically representing the meta-analysis of the malignant transformation of oral leukoplakia stratified by geographical region. Pooled proportions (expressed as percentage) and 95% confidence intervals (CI) were used as effect size metric.

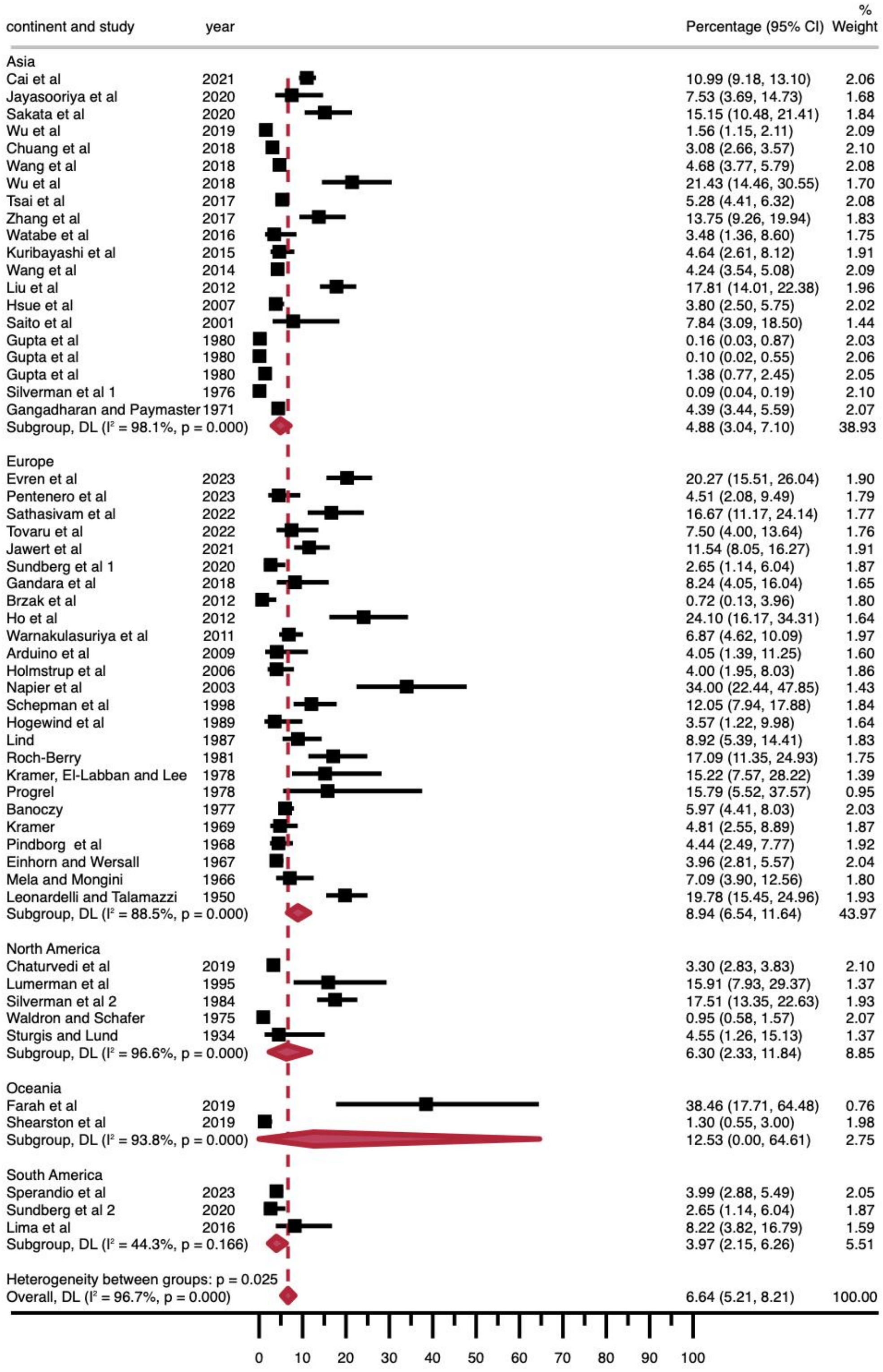

3.3 Subgroup meta-analysis stratified by source of patients

Figure S3. Forest plot graphically representing the meta-analysis of the malignant transformation of oral leukoplakia stratified by source of patients (population-based vs clinic-based studies). Pooled proportions (expressed as percentage) and 95% confidence intervals (CI) were used as effect size metric.

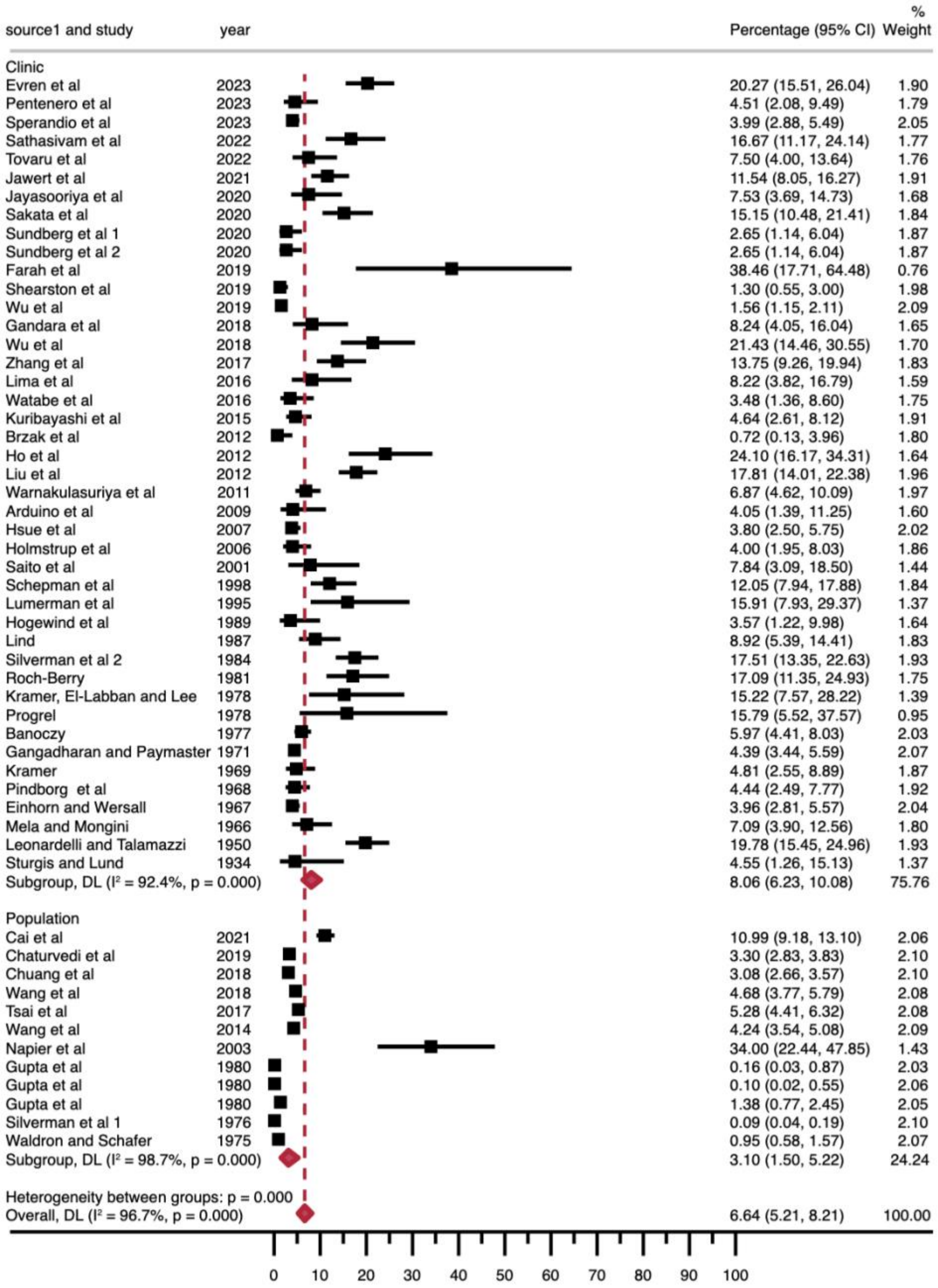

3.4 Subgroup meta-analysis stratified by study design

Figure S4. Forest plot graphically representing the meta-analysis of the malignant transformation of oral leukoplakia stratified by study design (prospective vs retrospective). Pooled proportions (expressed as percentage) and 95% confidence intervals (CI) were used as effect size metric.

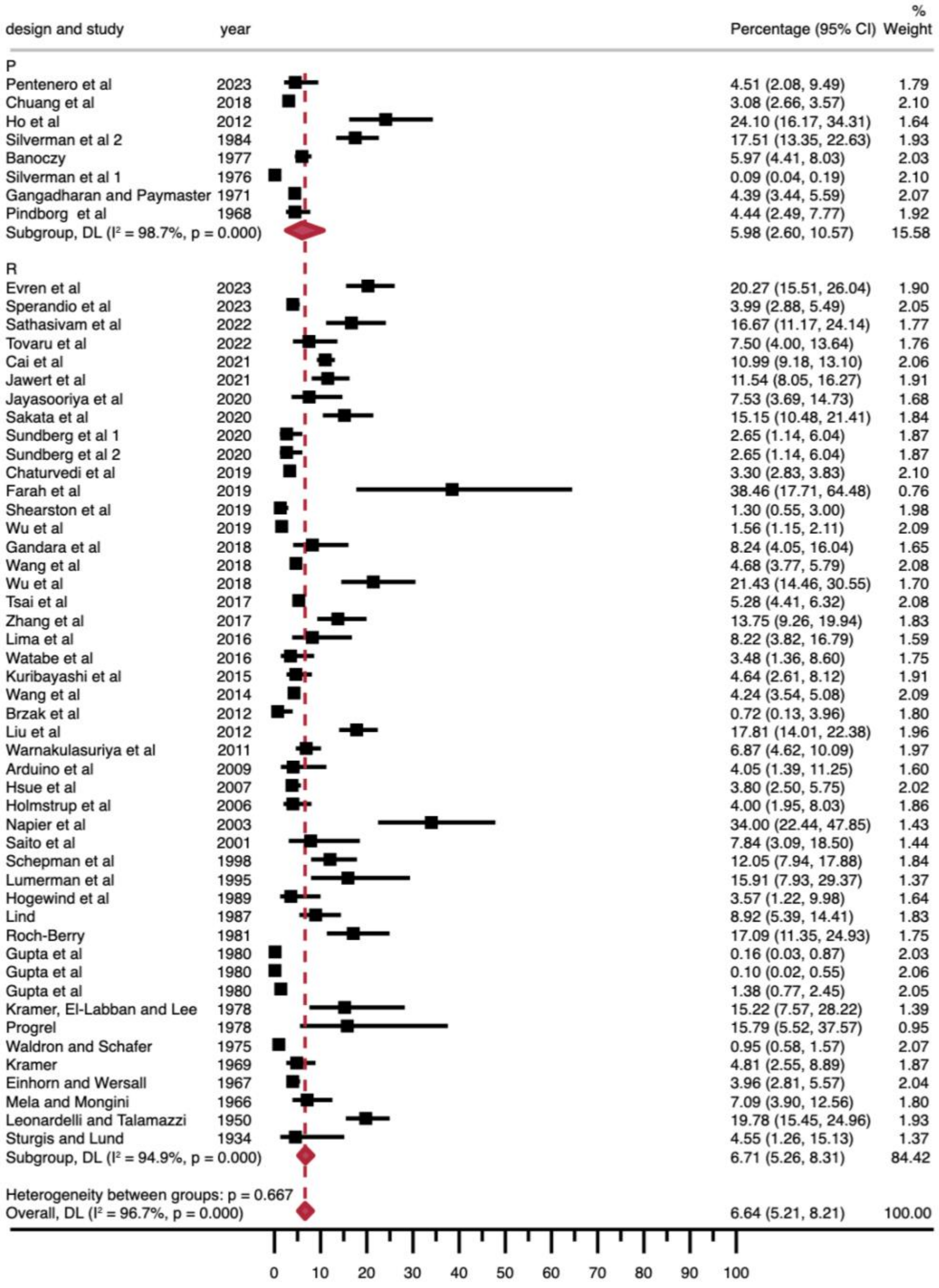

3.5. Subgroup meta-analysis stratified by site-distribution

Figure S5. Forest plot graphically representing the meta-analysis of the malignant transformation of oral leukoplakia stratified by site distribution oral cavity mixed vs tongue-floor of mouth. Pooled proportions (expressed as percentage) and 95% confidence intervals (CI) were used as effect size metric.

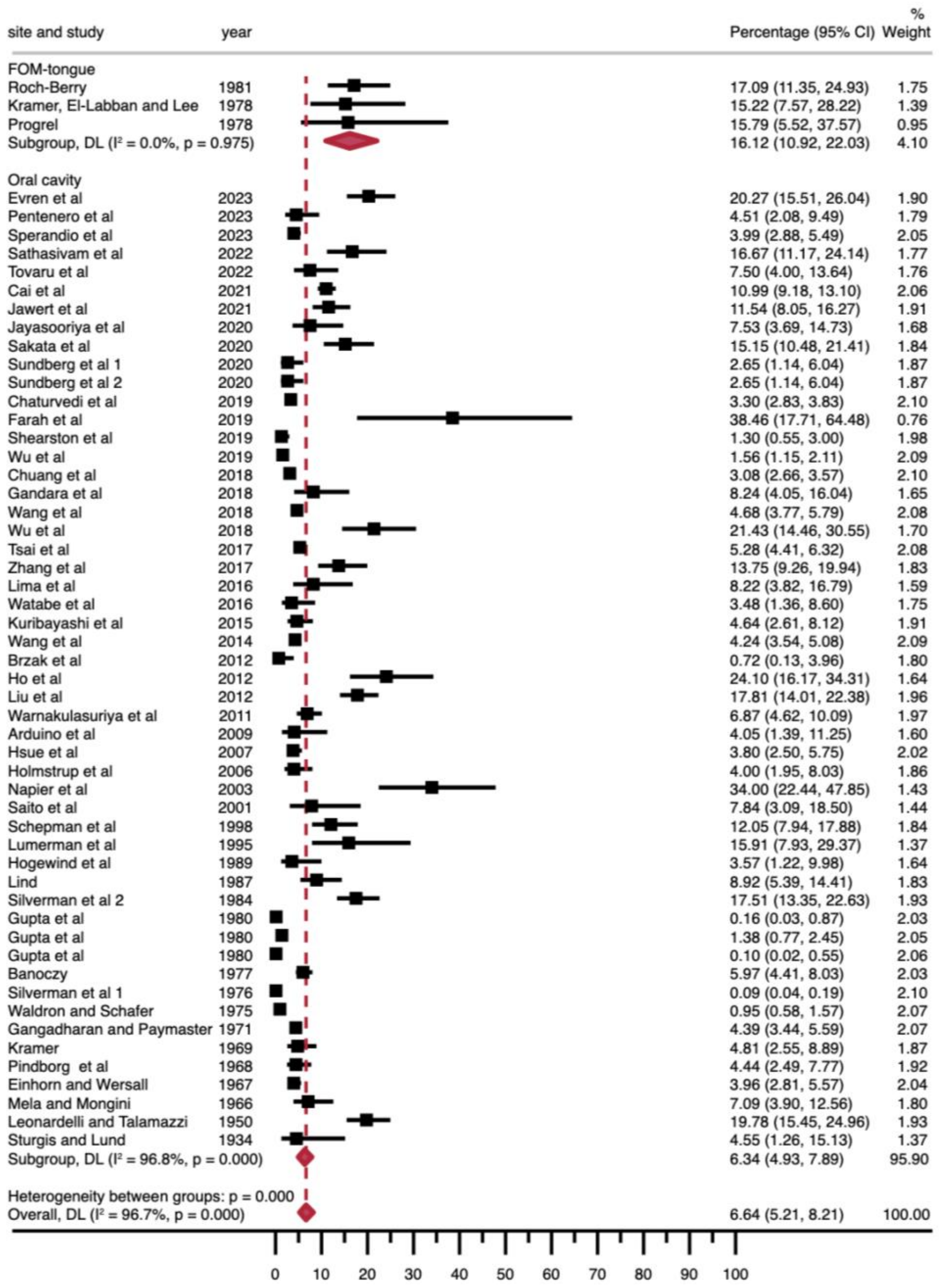

3.6. Sex and malignant transformation

Figure S6. Forest plot graphically representing meta-analysis of the malignant transformation of oral leukoplakia in females *versus* males with leukoplakia. Relative risk (RR) and 95% confidence intervals (CI) were used as effect size metric.

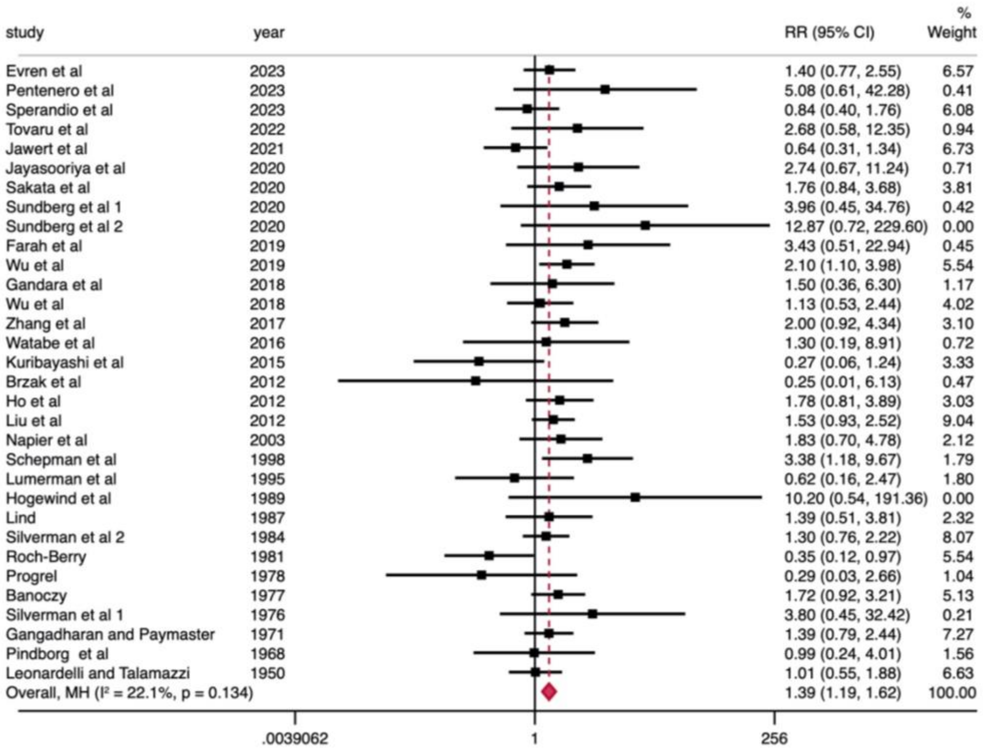

3.7. Age and malignant transformation

Figure S7. Forest plot graphically representing meta-analysis of the malignant transformation of oral leukoplakia in old *versus* young patients with leukoplakia. Relative risk (RR) and 95% confidence intervals (CI) were used as effect size metric.

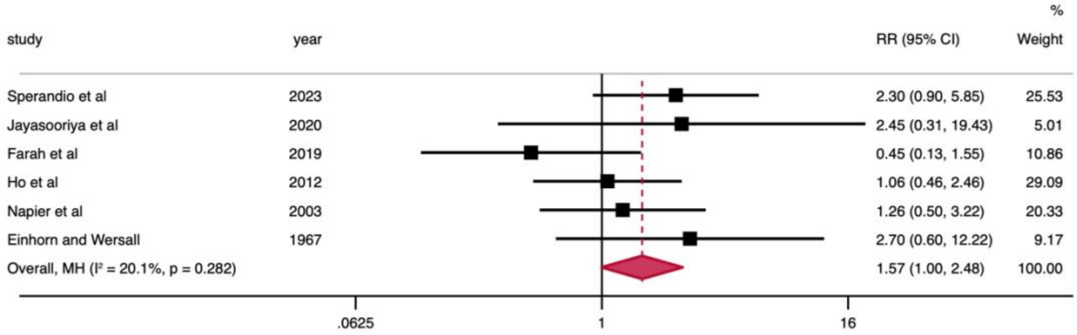

3.8. Tobacco and malignant transformation

Figure S8. Forest plot graphically representing meta-analysis of the malignant transformation of oral leukoplakia in smokers *versus* non-smokers patients with leukoplakia. Relative risk (RR) and 95% confidence intervals (CI) were used as effect size metric.

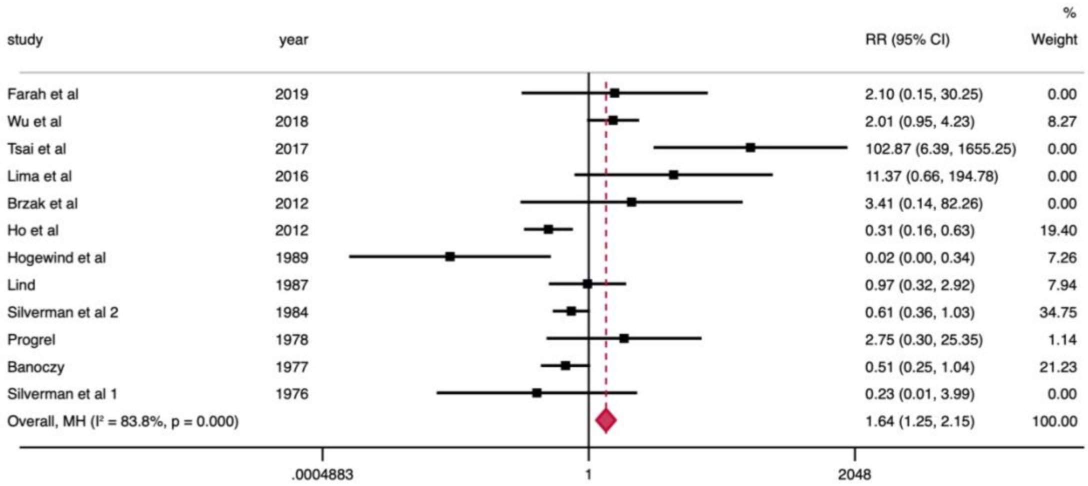

3.9. Alcohol and malignant transformation

Figure S9. Forest plot graphically representing meta-analysis of the malignant transformation of oral leukoplakia in drinkers *versus* non-drinkers patients with leukoplakia. Relative risk (RR) and 95% confidence intervals (CI) were used as effect size metric.

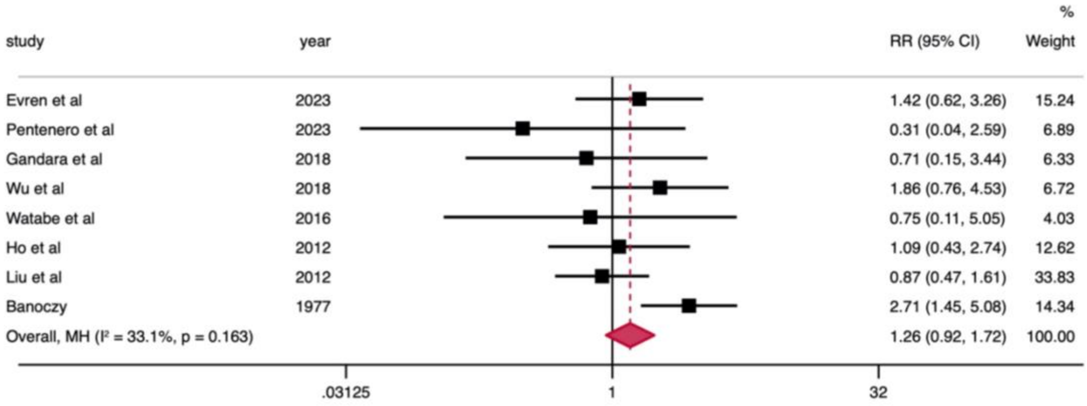

3.10. Betel quid and malignant transformation

Figure S10. Forest plot graphically representing meta-analysis of the malignant transformation of oral leukoplakia in betel quid chewers *versus* non-chewers patients with leukoplakia. Relative risk (RR) and 95% confidence intervals (CI) were used as effect size metric.

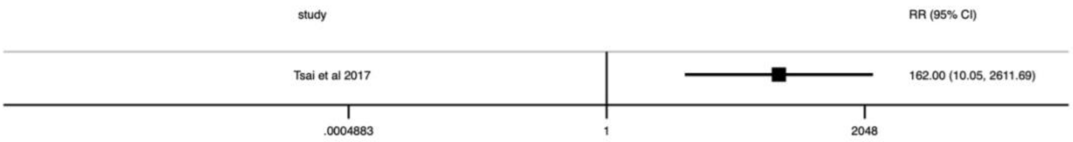

3.11. Size and malignant transformation

Figure S11. Forest plot graphically representing meta-analysis of the malignant transformation of oral leukoplakia according to size (large vs small) in patients with leukoplakia. Relative risk (RR) and 95% confidence intervals (CI) were used as effect size metric.

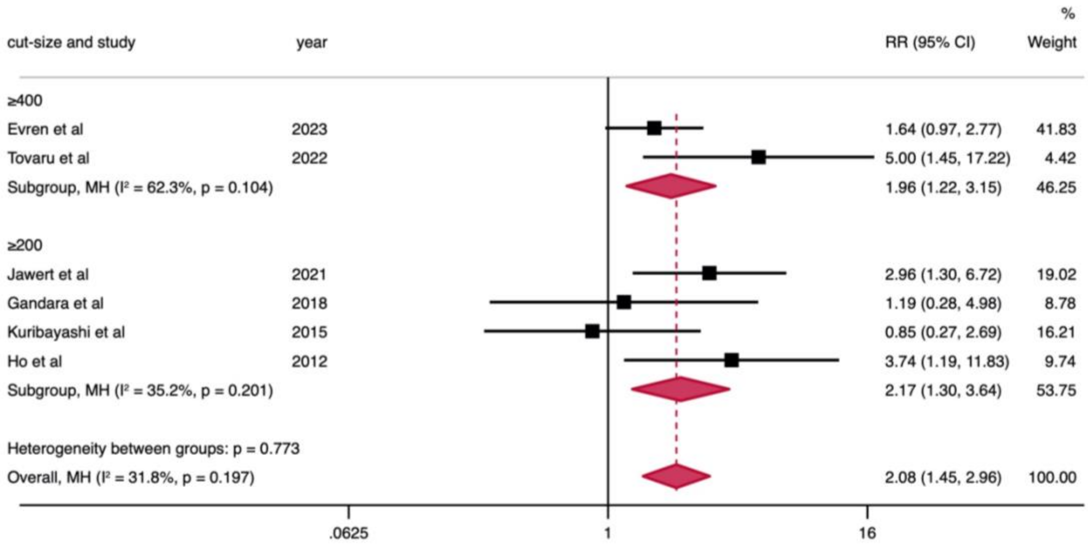

3.12. Clinical type and malignant transformation

Figure S12. Forest plot graphically representing meta-analysis of the malignant transformation of oral leukoplakia according to clinical type (non-homogeneous vs homogeneous vs homogeneous) in patients with leukoplakia. Relative risk (RR) and 95% confidence intervals (CI) were used as effect size metric.

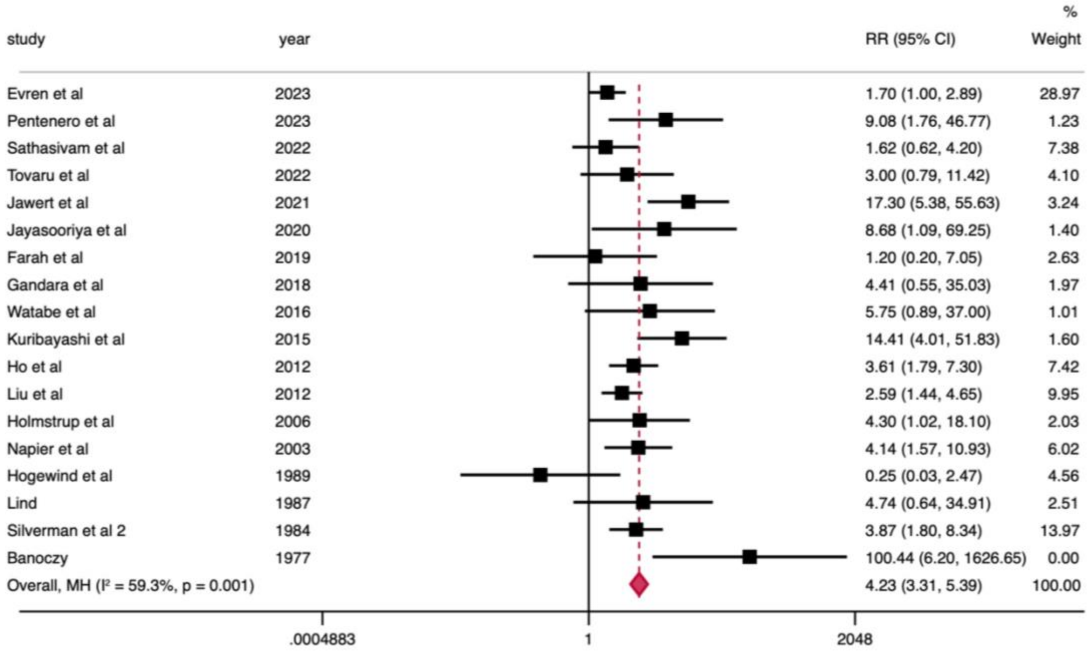

3.13. Malignant transformation of non-homogeneous leukoplakias

Figure S13. Forest plot graphically representing the meta-analysis of the malignant transformation of non-homogeneous oral leukoplakias. Pooled proportions (expressed as percentage) and 95% confidence intervals (CI) were used as effect size metric.

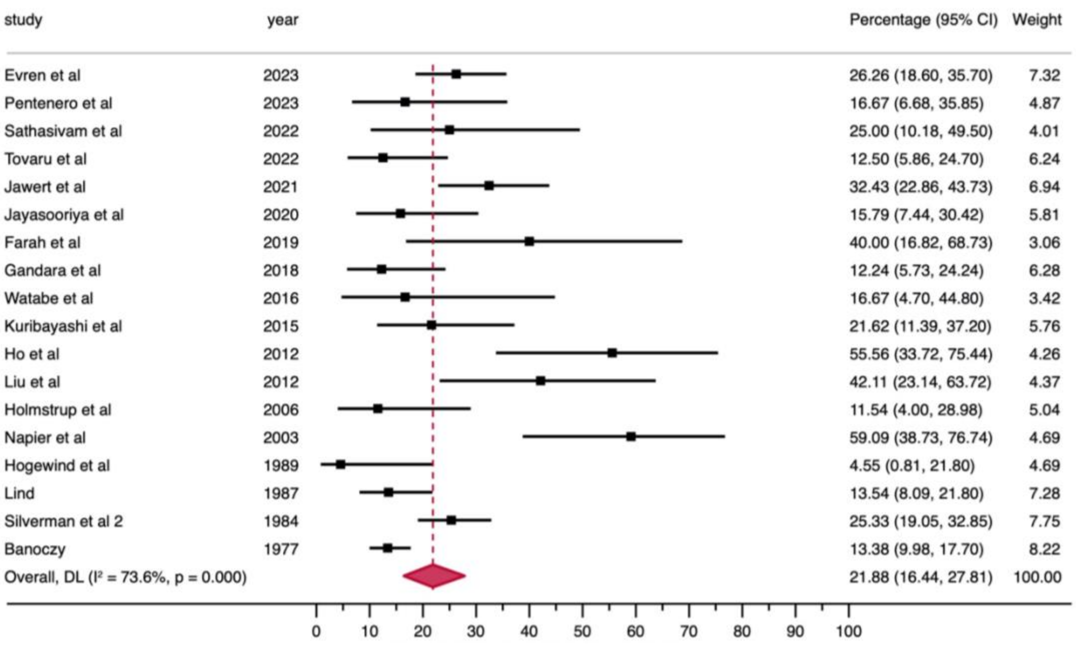

3.14. Malignant transformation of homogeneous leukoplakias

Figure S14. Forest plot graphically representing the meta-analysis of the malignant transformation of homogeneous oral leukoplakias. Pooled proportions (expressed as percentage) and 95% confidence intervals (CI) were used as effect size metric.

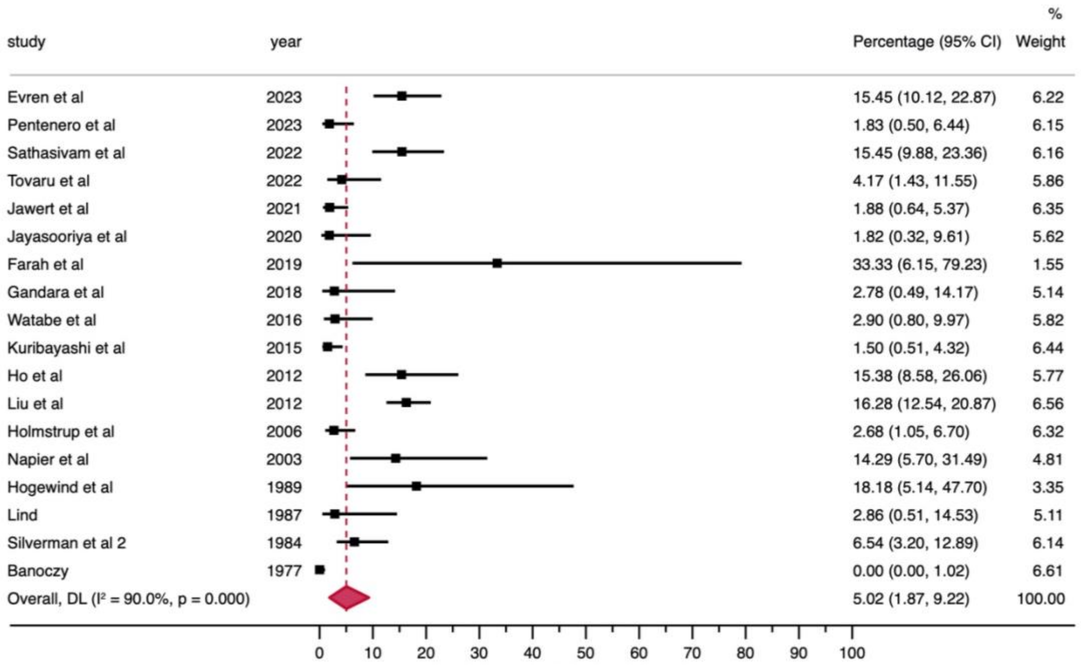

3.15. Location and malignant transformation

Figure S15. Forest plot graphically representing meta-analysis of the malignant transformation of oral leukoplakia according to the location (tongue vs others) in patients with oral leukoplakia. Relative risk (RR) and 95% confidence intervals (CI) were used as effect size metric.

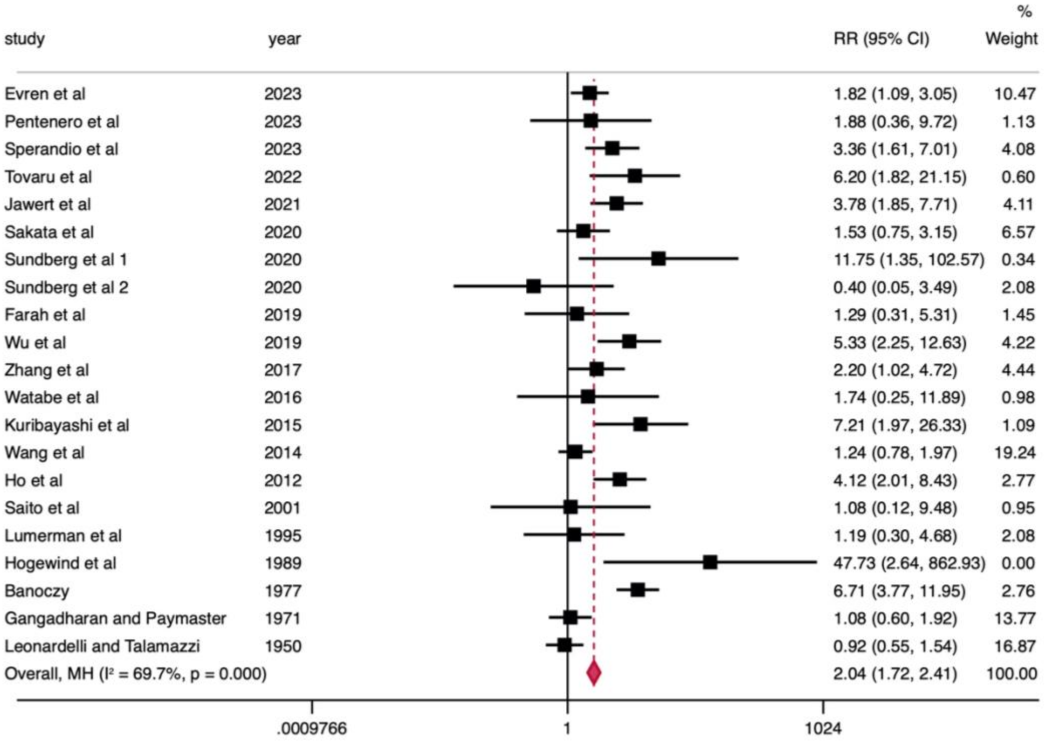

3.16. Tongue sublocation and malignant transformation

Figure S16. Forest plot graphically representing meta-analysis of the malignant transformation of oral leukoplakia according to tongue sublocation (border of tongue vs others) in patients with oral leukoplakia. Relative risk (RR) and 95% confidence intervals (CI) were used as effect size metric.

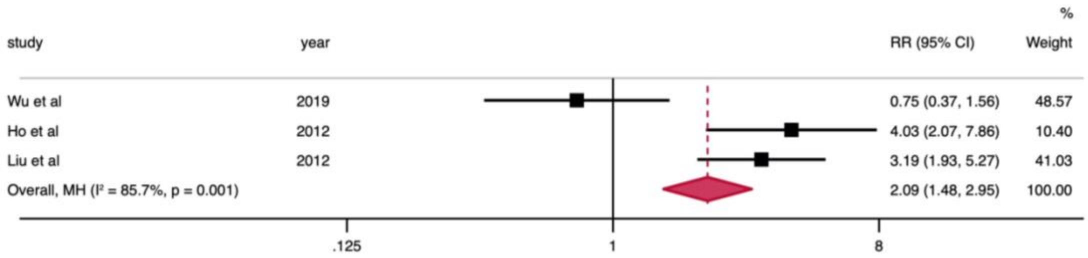

3.17. Malignant transformation of leukoplakias in tongue location

Figure S17. Forest plot graphically representing the meta-analysis of the malignant transformation of leukoplakias in tongue location. Pooled proportions (expressed as percentage) and 95% confidence intervals (CI) were used as effect size metric.

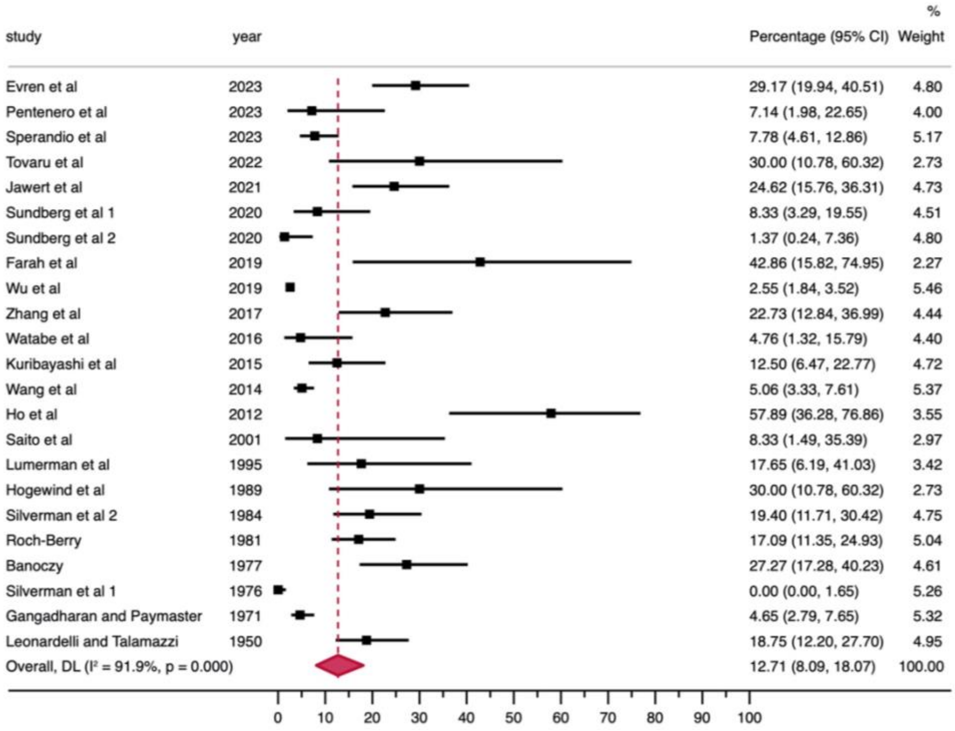

3.18. Malignant transformation of leukoplakias in buccal mucosa

Figure S18. Forest plot graphically representing the meta-analysis of the malignant transformation of leukoplakias in buccal mucosa. Pooled proportions (expressed as percentage) and 95% confidence intervals (CI) were used as effect size metric.

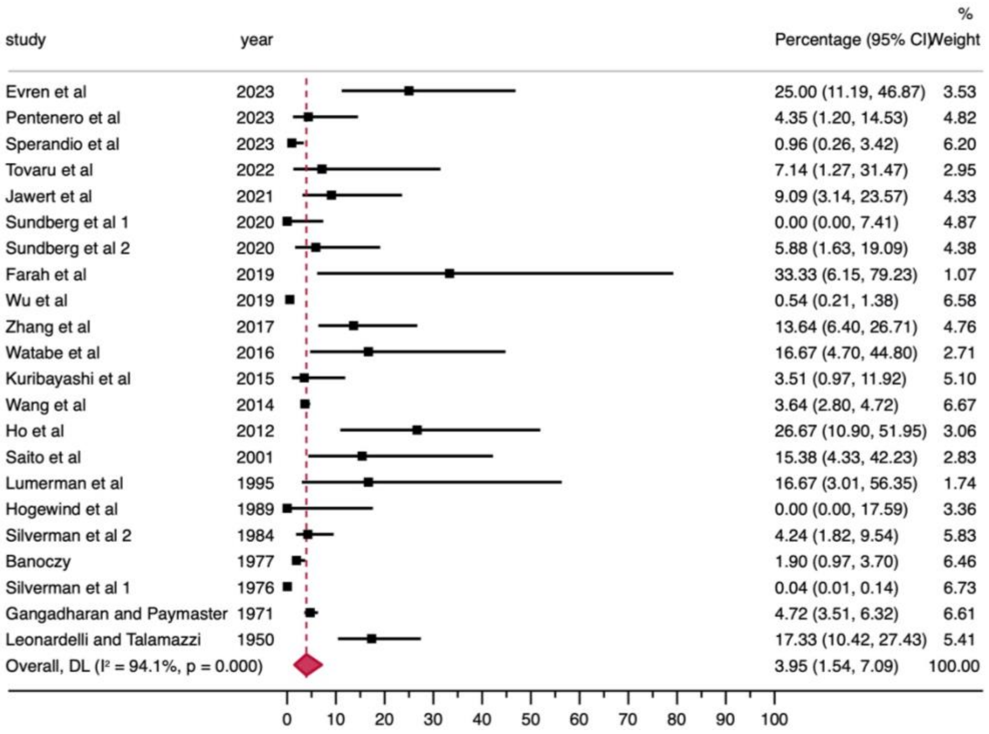

3.19. Malignant transformation of leukoplakias in floor of mouth

Figure S19. Forest plot graphically representing the meta-analysis of the malignant transformation of leukoplakias in floor of mouth. Pooled proportions (expressed as percentage) and 95% confidence intervals (CI) were used as effect size metric.

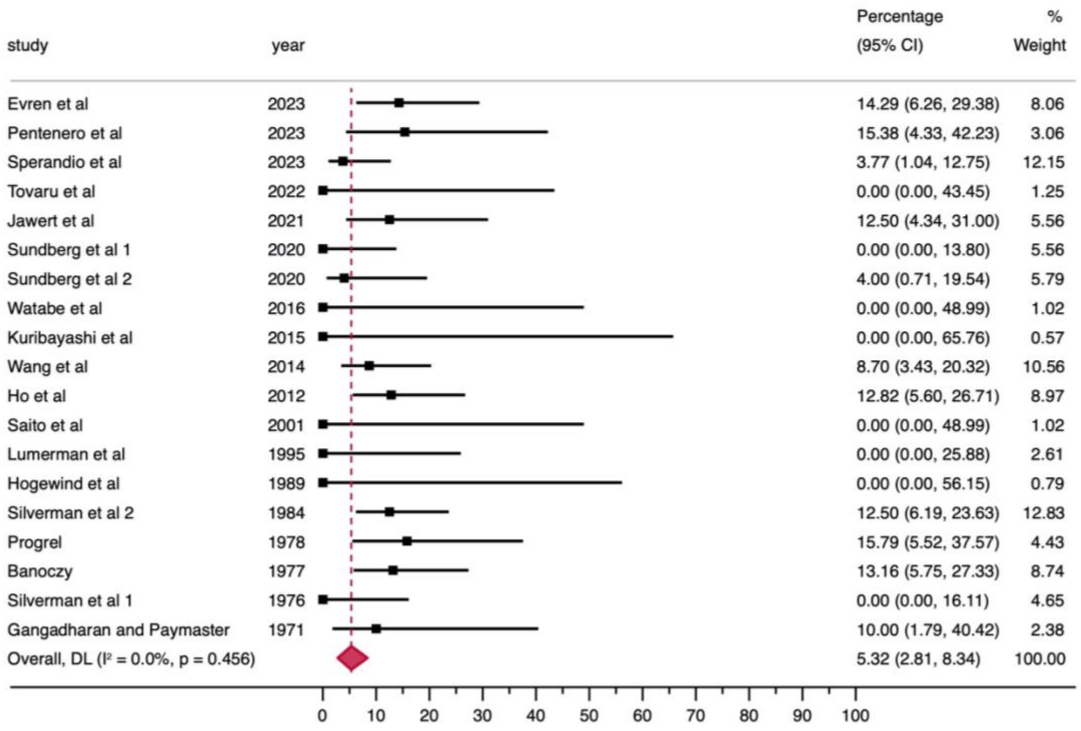

3.20. Malignant transformation of leukoplakias in palate

Figure S20. Forest plot graphically representing the meta-analysis of the malignant transformation of leukoplakias in palate. Pooled proportions (expressed as percentage) and 95% confidence intervals (CI) were used as effect size metric.

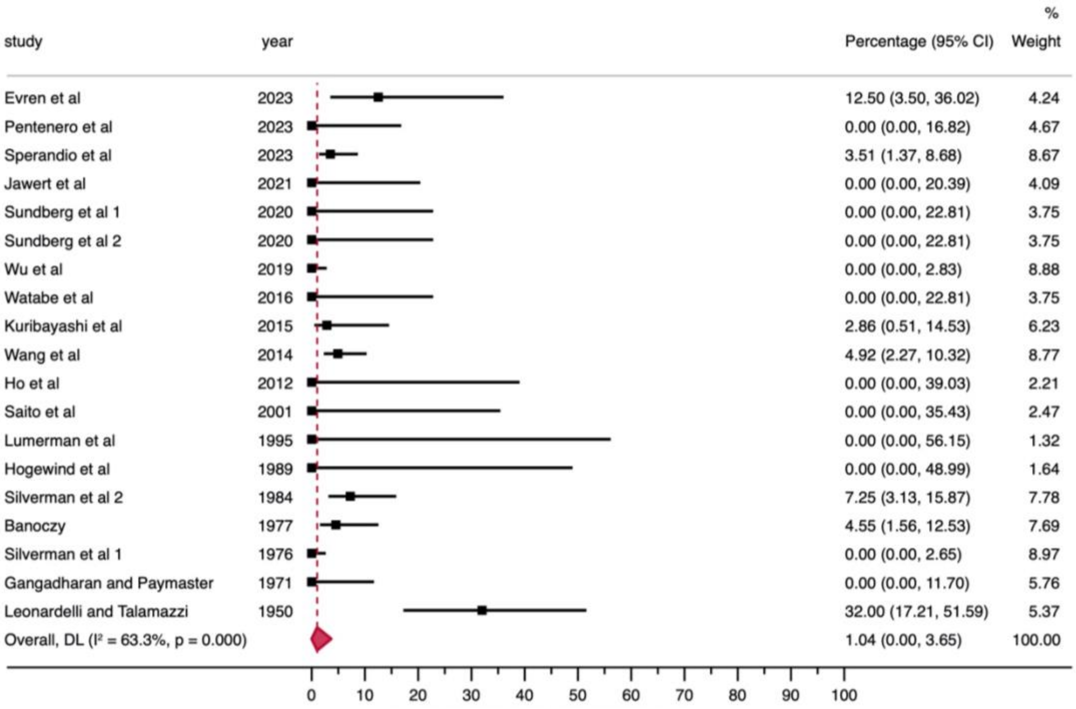

3.21. Malignant transformation of leukoplakias in lips

Figure S21. Forest plot graphically representing the meta-analysis of the malignant transformation of leukoplakias in lips. Pooled proportions (expressed as percentage) and 95% confidence intervals (CI) were used as effect size metric.

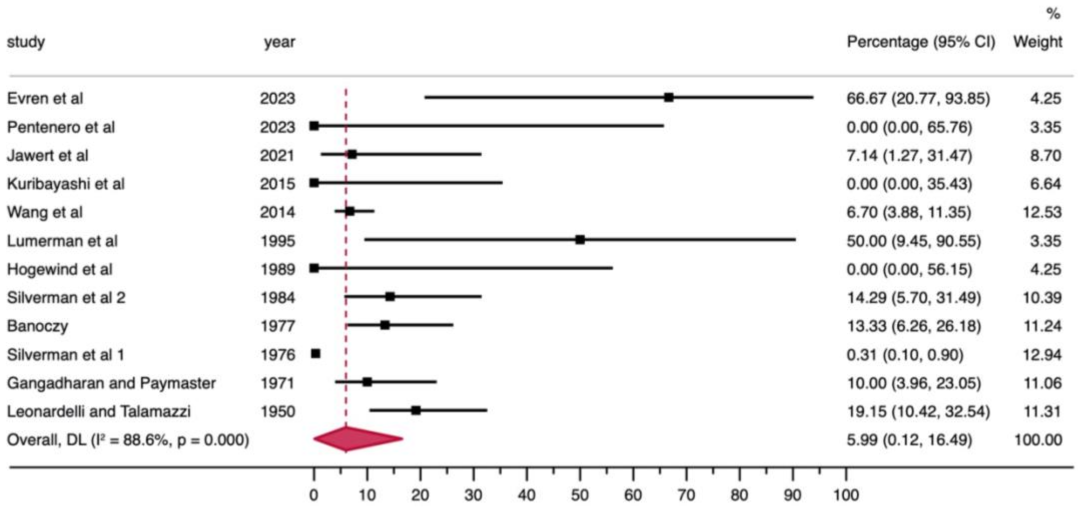

3.22. Malignant transformation of leukoplakias in retromolar trigone

Figure S22. Forest plot graphically representing the meta-analysis of the malignant transformation of leukoplakias in retromolar trigone. Pooled proportions (expressed as percentage) and 95% confidence intervals (CI) were used as effect size metric.

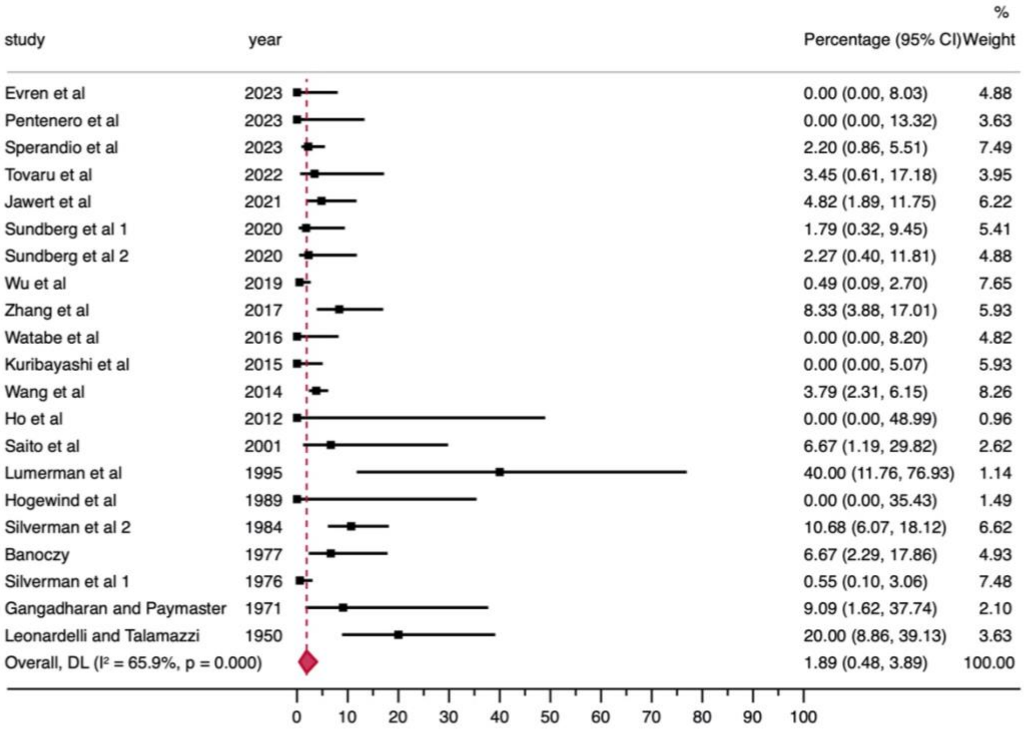

3.23. Malignant transformation of leukoplakias in lips

Figure S23. Forest plot graphically representing the meta-analysis of the malignant transformation of leukoplakias in retromolar trigone. Pooled proportions (expressed as percentage) and 95% confidence intervals (CI) were used as effect size metric.

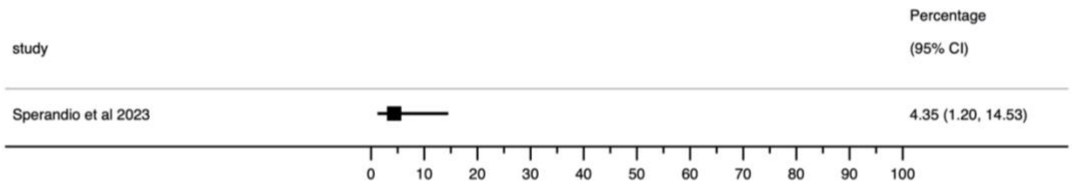

3.24. Presence of oral epithelial dysplasia and malignant transformation

Figure S24. Forest plot graphically representing meta-analysis of the malignant transformation of oral leukoplakia according to the presence of oral epithelial dysplasia (presence vs absence) in patients with leukoplakia. Relative risk (RR) and 95% confidence intervals (CI) were used as effect size metric.

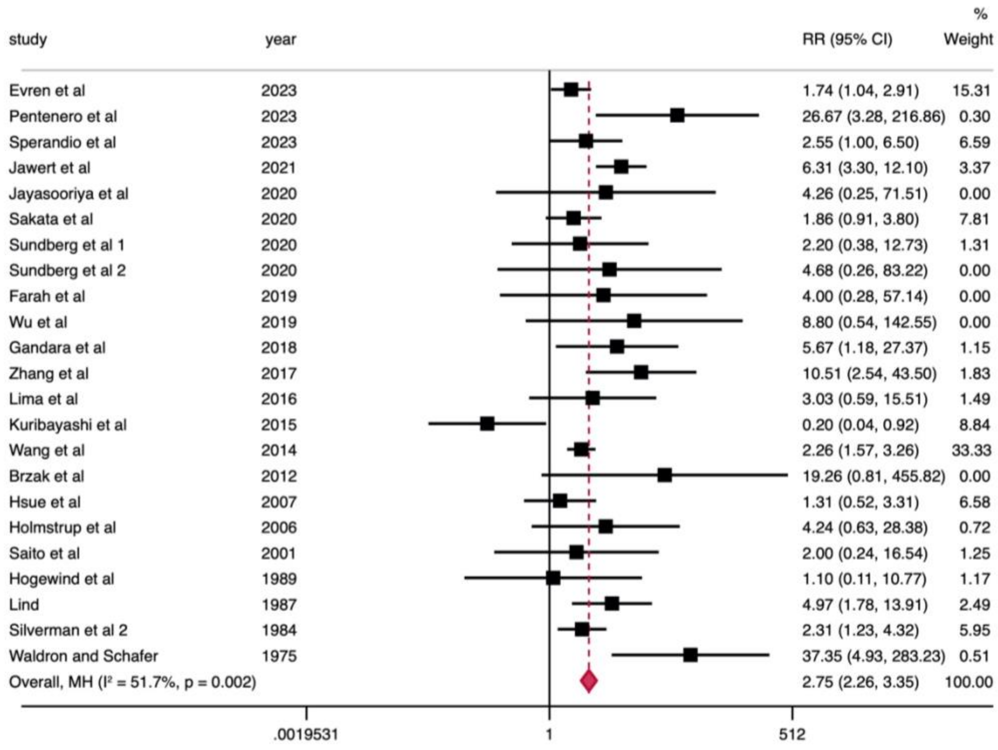

3.25. Grade oral epithelial dysplasia and malignant transformation

Figure S25. Forest plot graphically representing meta-analysis of the malignant transformation of oral leukoplakia according to the grade of oral epithelial dysplasia (high grade vs low grade) in patients with leukoplakia. Relative risk (RR) and 95% confidence intervals (CI) were used as effect size metric.

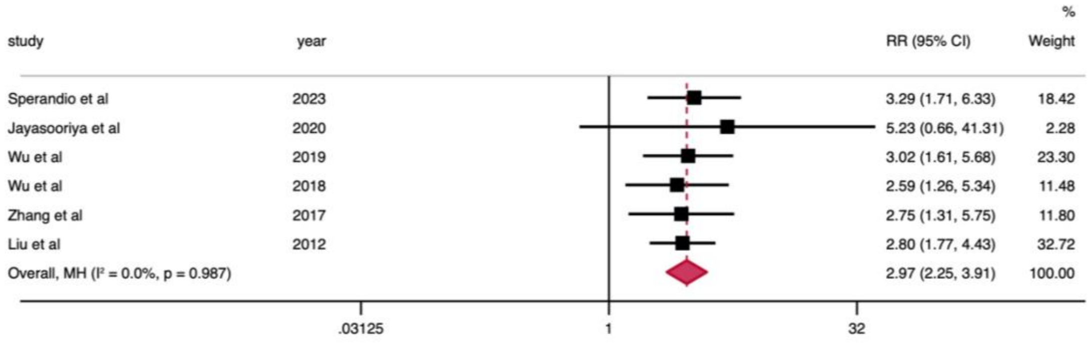

3.26. Malignant transformation of leukoplakias and dysplasia

Figure S26a. Forest plot graphically representing the meta-analysis of the malignant transformation of oral leukoplakia without dysplasia. Pooled proportions (expressed as percentage) and 95% confidence intervals (CI) were used as effect size metric.

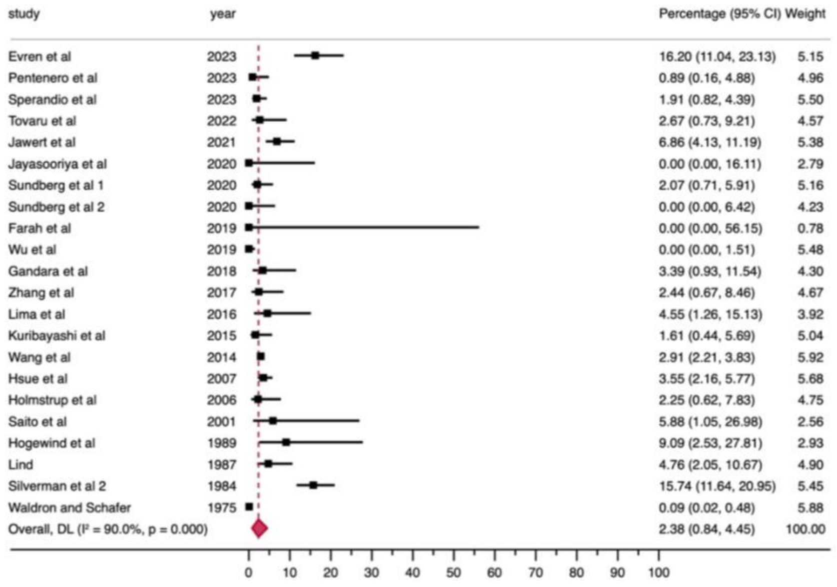

Figure S26b. Forest plot graphically representing the meta-analysis of the malignant transformation of oral leukoplakia with mild dysplasia. Pooled proportions (expressed as percentage) and 95% confidence intervals (CI) were used as effect size metric.

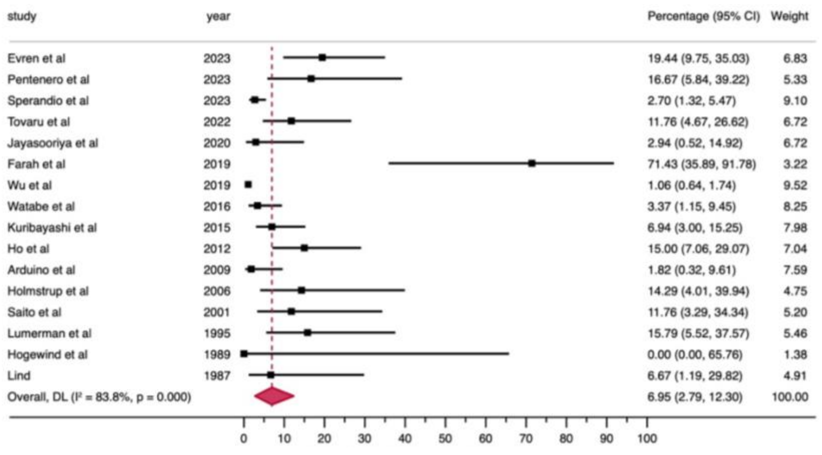

Figure S26c. Forest plot graphically representing the meta-analysis of the malignant transformation of oral leukoplakia with moderate dysplasia. Pooled proportions (expressed as percentage) and 95% confidence intervals (CI) were used as effect size metric.

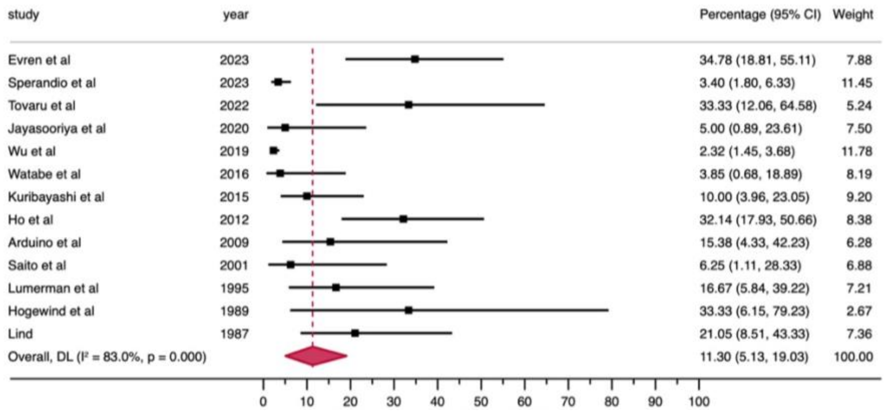

Figure S26d. Forest plot graphically representing the meta-analysis of the malignant transformation of oral leukoplakia with severe dysplasia. Pooled proportions (expressed as percentage) and 95% confidence intervals (CI) were used as effect size metric.

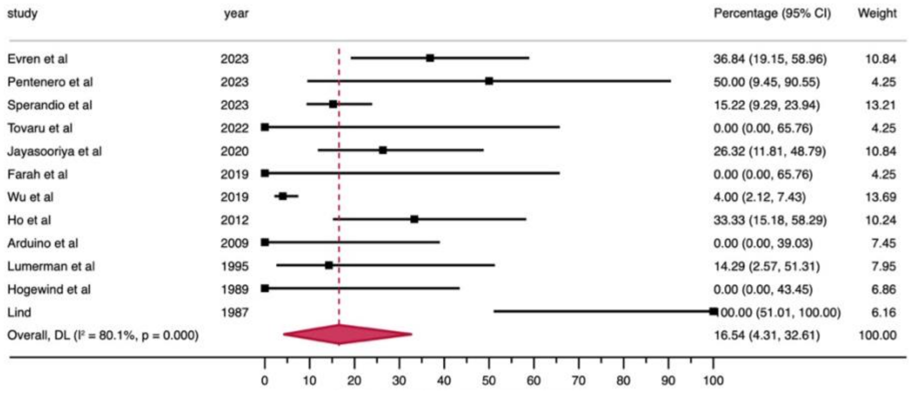

4. Sensitivity analysis

Figure S27. Sensitivity analysis (leave-one-out method) of the pooled studies in the meta-analysis on the malignant transformation of oral leukoplakia.

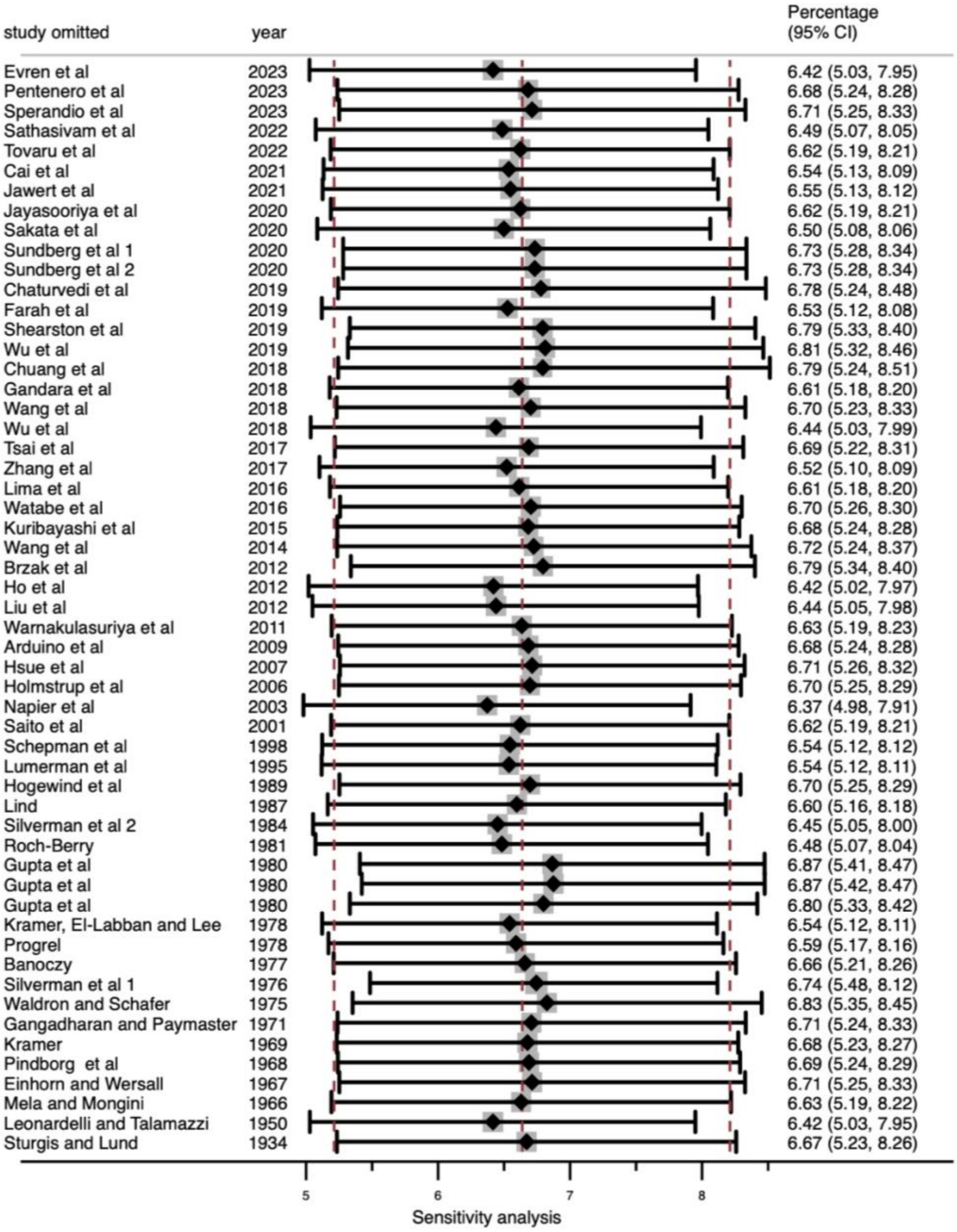

5. Analysis of small-study effects.

Figure S28. A funnel plot of estimated transformed proportions against their standard errors, graphically representing the analysis of "small-study" effects on the malignant transformation of oral leukoplakia. The black vertical line corresponds to the pooled estimated transformed proportion. The two diagonal intermittent lines represent the pseudo-95% confidence interval. The blue circles represent the published studies.

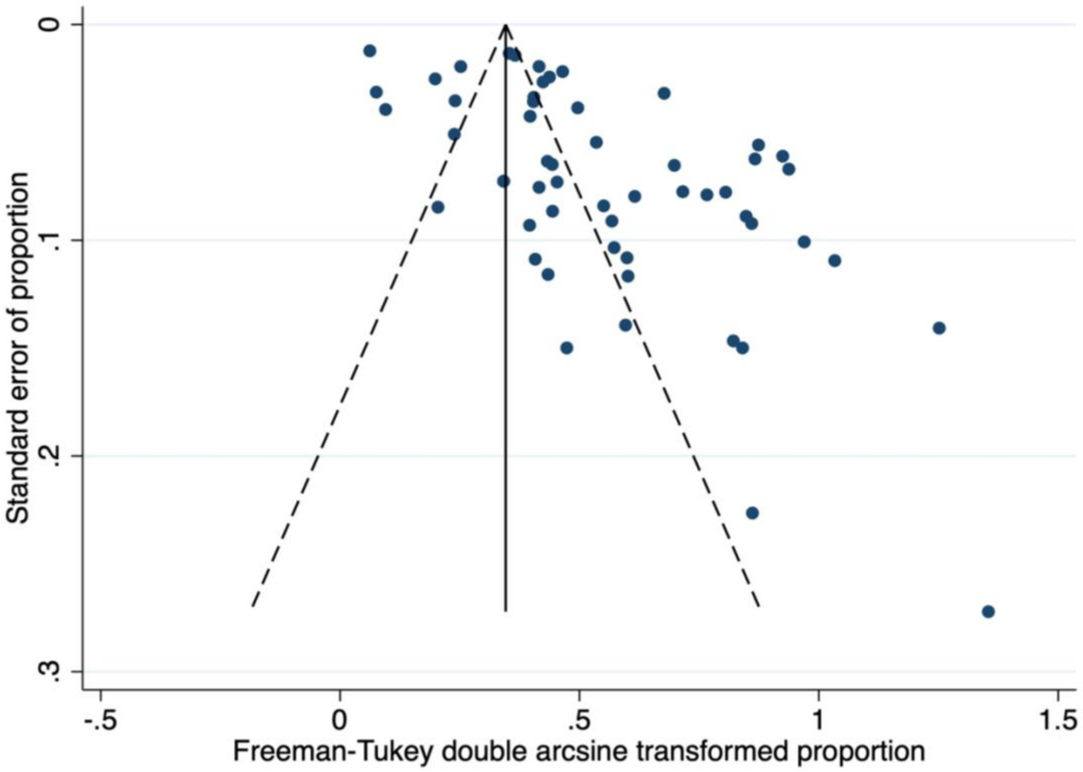

6. List of included studies (List S1)

Arduino, P. G., Surace, A., Carbone, M., Elia, A., Massolini, G., Gandolfo, S., & Broccoletti, R. (2009). Outcome of oral dysplasia: a retrospective hospital-based study of 207 patients with a long follow-up. *Journal of oral pathology & medicine : official publication of the International Association of Oral Pathologists and the American Academy of Oral Pathology*, *38*(6), 540–544. https://doi.org/10.1111/j.1600-0714.2009.00782.x

Bánóczy J. (1977). Follow-up studies in oral leukoplakia. *Journal of maxillofacial surgery*, *5*(1), 69–75. https://doi.org/10.1016/s0301-0503(77)80079-9

Brzak, B. L., Mravak-Stipetić, M., Canjuga, I., Baricević, M., Balicević, D., Sikora, M., & Filipović-Zore, I. (2012). The frequency and malignant transformation rate of oral lichen planus and leukoplakia--a retrospective study. *Collegium antropologicum*, *36*(3), 773–777.

Cai, X., Zhang, J., Han, Y., Tang, Q., Zhang, H., & Li, T. (2021). Development and validation of a nomogram prediction model for malignant transformation of oral potentially malignant disorders. *Oral oncology*, *123*, 105619. https://doi.org/10.1016/j.oraloncology.2021.105619

Chaturvedi, A. K., Udaltsova, N., Engels, E. A., Katzel, J. A., Yanik, E. L., Katki, H. A., Lingen, M. W., & Silverberg, M. J. (2020). Oral Leukoplakia and Risk of Progression to Oral Cancer: A Population-Based Cohort Study. *Journal of the National Cancer Institute*, *112*(10), 1047–1054. https://doi.org/10.1093/jnci/djz238

Chuang, S. L., Wang, C. P., Chen, M. K., Su, W. W., Su, C. W., Chen, S. L., Chiu, S. Y., Fann, J. C., & Yen, A. M. (2018). Malignant transformation to oral cancer by subtype of oral potentially malignant disorder: A prospective cohort study of Taiwanese nationwide oral cancer screening program. *Oral oncology*, *87*, 58–63. https://doi.org/10.1016/j.oraloncology.2018.10.021

Einhorn, J., & Wersall, J. (1967). Incidence of oral carcinoma in patients with leukoplakia of the oral mucosa. *Cancer*, *20*(12), 2189–2193. https://doi.org/10.1002/1097-0142(196712)20:12<2189::aid-cncr2820201218>3.0.co;2-m

Evren, I., Najim, A. M., Poell, J. B., Brouns, E. R., Wils, L. J., Peferoen, L. A. N., Brakenhoff, R. H., Bloemena, E., van der Meij, E. H., & de Visscher, J. G. A. M. (2023). The value of regular follow-up of oral leukoplakia for early detection of malignant transformation. *Oral diseases*, 10.1111/odi.14797. Advance online publication. https://doi.org/10.1111/odi.14797

Farah, C. S., Jessri, M., Bennett, N. C., Dalley, A. J., Shearston, K. D., & Fox, S. A. (2019). Exome sequencing of oral leukoplakia and oral squamous cell carcinoma implicates DNA damage repair gene defects in malignant transformation. *Oral*

oncology, *96*, 42–50. https://doi.org/10.1016/j.oraloncology.2019.07.005

Gandara-Vila, P., Perez-Sayans, M., Suarez-Penaranda, J. M., Gallas-Torreira, M., Somoza-Martin, J., Reboiras-Lopez, M. D., Blanco-Carrion, A., & Garcia-Garcia, A. (2018). Survival study of leukoplakia malignant transformation in a region of northern Spain. *Medicina oral, patologia oral y cirugia bucal*, *23*(4), e413–e420. https://doi.org/10.4317/medoral.22326

Gangadharan, P., & Paymaster, J. C. (1971). Leukoplakia--an epidemiologic study of 1504 cases observed at the Tata Memorial Hospital, Bombay, India. *British journal of cancer*, *25*(4), 657–668. https://doi.org/10.1038/bjc.1971.81

Gupta, P. C., Mehta, F. S., Daftary, D. K., Pindborg, J. J., Bhonsle, R. B., Jalnawalla, P. N., Sinor, P. N., Pitkar, V. K., Murti, P. R., Irani, R. R., Shah, H. T., Kadam, P. M., Iyer, K. S., Iyer, H. M., Hegde, A. K., Chandrashekar, G. K., Shiroff, B. C., Sahiar, B. E., & Mehta, M. N. (1980). Incidence rates of oral cancer and natural history of oral precancerous lesions in a 10-year follow-up study of Indian villagers. *Community dentistry and oral epidemiology*, *8*(6), 283–333. https://doi.org/10.1111/j.1600-0528.1980.tb01302.x

Ho, M. W., Risk, J. M., Woolgar, J. A., Field, E. A., Field, J. K., Steele, J. C., Rajlawat, B. P., Triantafyllou, A., Rogers, S. N., Lowe, D., & Shaw, R. J. (2012). The clinical determinants of malignant transformation in oral epithelial dysplasia. *Oral oncology*, *48*(10), 969–976. https://doi.org/10.1016/j.oraloncology.2012.04.002

Hogewind, W. F., van der Kwast, W. A., & van der Waal, I. (1989). Oral leukoplakia, with emphasis on malignant transformation. A follow-up study of 46 patients. *Journal of cranio-maxillo-facial surgery : official publication of the European Association for Cranio-Maxillo-Facial Surgery*, *17*(3), 128–133. https://doi.org/10.1016/s1010-5182(89)80085-x

Holmstrup, P., Vedtofte, P., Reibel, J., & Stoltze, K. (2006). Long-term treatment outcome of oral premalignant lesions. *Oral oncology*, *42*(5), 461–474. https://doi.org/10.1016/j.oraloncology.2005.08.011

Hsue, S. S., Wang, W. C., Chen, C. H., Lin, C. C., Chen, Y. K., & Lin, L. M. (2007). Malignant transformation in 1458 patients with potentially malignant oral mucosal disorders: a follow-up study based in a Taiwanese hospital. *Journal of oral pathology & medicine : official publication of the International Association of Oral Pathologists and the American Academy of Oral Pathology*, *36*(1), 25–29. https://doi.org/10.1111/j.1600-0714.2006.00491.x

Jäwert, F., Pettersson, H., Jagefeldt, E., Holmberg, E., Kjeller, G., & Öhman, J. (2021). Clinicopathologic factors associated with malignant transformation of oral leukoplakias: a retrospective cohort study. *International journal of oral and maxillofacial surgery*, *50*(11), 1422–1428. https://doi.org/10.1016/j.ijom.2021.01.012

Jayasooriya, P. R., Dayaratne, K., Dissanayake, U. B., & Warnakulasuriya, S. (2020). Malignant transformation of oral leukoplakia: a follow-up study. *Clinical oral investigations*, *24*(12), 4563–4569. https://doi.org/10.1007/s00784-020-03322-4

Kramer I. R. (1969). Precancerous conditions of the oral mucosa. A computer-aided study. *Annals of the Royal College of Surgeons of England*, *45*(6), 340–356.

Kramer, I. R., El-Labban, N., & Lee, K. W. (1978). The clinical features and risk of malignant transformation in sublingual keratosis. *British dental journal*, *144*(6), 171–180. https://doi.org/10.1038/sj.bdj.4804055

Kuribayashi, Y., Tsushima, F., Morita, K. I., Matsumoto, K., Sakurai, J., Uesugi, A., Sato, K., Oda, S., Sakamoto, K., & Harada, H. (2015). Long-term outcome of non-surgical treatment in patients with oral leukoplakia. *Oral oncology*, *51*(11), 1020–1025. https://doi.org/10.1016/j.oraloncology.2015.09.004

Leonardelli, G. B., & Talamazzi, F. (1950). Leucoplasie del cavo orale e precancerosi [Leukoplakia of the oral cavity and precancerous conditions]. *Archivio italiano di otologia, rinologia e laringologia*, *61*(2), 107–114.

Lima, J. S., Correa, L., Klingbeil, M. F., & de Sousa, S. C. (2016). c-Jun, pc-Jun, and p27 are differently expressed in oral leukoplakias in smokers and never-smokers. *Oral surgery, oral medicine, oral pathology and oral radiology*, *121*(1), 73–80. https://doi.org/10.1016/j.oooo.2015.09.003

Lind P. O. (1987). Malignant transformation in oral leukoplakia. *Scandinavian journal of dental research*, *95*(6), 449–455. https://doi.org/10.1111/j.1600-0722.1987.tb01959.x

Liu, W., Shi, L. J., Wu, L., Feng, J. Q., Yang, X., Li, J., Zhou, Z. T., & Zhang, C. P. (2012). Oral cancer development in patients with leukoplakia--clinicopathological factors affecting outcome. *PloS one*, *7*(4), e34773. https://doi.org/10.1371/journal.pone.0034773

Lumerman, H., Freedman, P., & Kerpel, S. (1995). Oral epithelial dysplasia and the development of invasive squamous cell carcinoma. *Oral surgery, oral medicine, oral pathology, oral radiology, and endodontics*, *79*(3), 321–329. https://doi.org/10.1016/s1079-2104(05)80226-4

Mela, F., & Mongini, F. (1966). Contributo casistico allo studio delle leucoplachie orali. Indagine clinica di controllo su 141 casi sottoposti a biopsia [Case contribution to the study of oral leukoplakias. (Clinical follow up of 141 biopsied cases)]. *Minerva stomatologica*, *15*(7), 502–507.

Napier, S. S., Cowan, C. G., Gregg, T. A., Stevenson, M., Lamey, P. J., & Toner, P. G. (2003). Potentially malignant oral lesions in Northern Ireland: size (extent) matters. *Oral diseases*, *9*(3), 129–137. https://doi.org/10.1034/j.1601-0825.2003.02888.x

Pentenero, M., Castagnola, P., Castillo, F. V., Isaevska, E., Sutera, S., & Gandolfo, S. (2023). Predictors of malignant transformation in oral leukoplakia and proliferative verrucous leukoplakia: An observational prospective study including the DNA ploidy status. *Head & neck*, *45*(10), 2589–2604. https://doi.org/10.1002/hed.27483

Pindborg, J. J., Jolst, O., Renstrup, G., & Roed-Petersen, B. (1968). Studies in oral leukoplakia: a preliminary report on the period pervalence of malignant transformation in leukoplakia based on a follow-up study of 248 patients. *Journal of the American Dental Association (1939)*, *76*(4), 767–771. https://doi.org/10.14219/jada.archive.1968.0127

Pogrel M. A. (1979). Sublingual keratosis and malignant transformation. *Journal of oral pathology*, *8*(3), 176–178. https://doi.org/10.1111/j.1600-0714.1979.tb01824.x

Roch-Berry C. S. (1981). Malignant changes in glossal leukoplakia. *Clinical radiology*, *32*(6), 693–694. https://doi.org/10.1016/s0009-9260(81)80341-8

Saito, T., Sugiura, C., Hirai, A., Notani, K., Totsuka, Y., Shindoh, M., & Fukuda, H. (2001). Development of squamous cell carcinoma from pre-existent oral leukoplakia: with respect to treatment modality. *International journal of oral and maxillofacial surgery*, *30*(1), 49–53. https://doi.org/10.1054/ijom.2000.0012

Sathasivam, H. P., Sloan, P., Thomson, P. J., & Robinson, M. (2022). The clinical utility of contemporary oral epithelial dysplasia grading systems. *Journal of oral pathology & medicine : official publication of the International Association of Oral Pathologists and the American Academy of Oral Pathology*, *51*(2), 180–187. https://doi.org/10.1111/jop.13262

Sakata, J., Yoshida, R., Matsuoka, Y., Kawahara, K., Arita, H., Nakashima, H., et al. (2020). FOXP3 lymphocyte status may predict the risk of malignant transformation in oral leukoplakia. *Journal of Oral and Maxillofacial Surgery, Medicine, and Pathology*, *32*(1), 33–39. https://doi.org/10.1016/j.ajoms.2019.06.005

Schepman, K. P., van der Meij, E. H., Smeele, L. E., & van der Waal, I. (1998). Malignant transformation of oral leukoplakia: a follow-up study of a hospital-based population of 166 patients with oral leukoplakia from The Netherlands. *Oral oncology*, *34*(4), 270–275.

Shearston, K., Fateh, B., Tai, S., Hove, D., & Farah, C. S. (2019). Malignant transformation rate of oral leukoplakia in an Australian population. *Journal of oral pathology & medicine : official publication of the International Association of Oral Pathologists and the American Academy of Oral Pathology*, *48*(7), 530–537. https://doi.org/10.1111/jop.12899

Silverman, S., Bhargava, K., Smith, L. W., & Malaowalla, A. M. (1976). Malignant transformation and natural history of oral leukoplakia in 57,518 industrial workers

of Gujarat, India. *Cancer*, *38*(4), 1790–1795. https://doi.org/10.1002/1097-0142(197610)38:4<1790::aid-cncr2820380456>3.0.co;2-i

Silverman, S., Jr, Gorsky, M., & Lozada, F. (1984). Oral leukoplakia and malignant transformation. A follow-up study of 257 patients. *Cancer*, *53*(3), 563–568. https://doi.org/10.1002/1097-0142(19840201)53:3<563::aid-cncr2820530332>3.0.co;2-f

Sperandio, M., Warnakulasuriya, S., Soares, A. B., Passador-Santos, F., Mariano, F. V., Lima, C. S. P., Scarini, J. F., Dominguete, M. H. L., de Camargo Moraes, P., Montalli, V. A. M., Hellmeister, L., & de Araújo, V. C. (2023). Oral epithelial dysplasia grading: Comparing the binary system to the traditional 3-tier system, an actuarial study with malignant transformation as outcome. *Journal of oral pathology & medicine : official publication of the International Association of Oral Pathologists and the American Academy of Oral Pathology*, *52*(5), 418–425. https://doi.org/10.1111/jop.13365

Sturgis, S.H., and Lund, C.C. (1934). Leukoplakia buccalis and keratosis labialis. *New Eng J Med , 210, 996.*

Sundberg, J., Öhman, J., Korytowska, M., Wallström, M., Kjeller, G., Andersson, M., Horal, P., Lindh, M., Giglio, D., Kovács, A., Sand, L., Hirsch, J. M., Magda Araújo Ferracini, L., de Souza, A. C. M. F., Parlatescu, I., Dobre, M., Hinescu, M. E., Braz-Silva, P. H., Tovaru, S., & Hasséus, B. (2021). High-risk human papillomavirus in patients with oral leukoplakia and oral squamous cell carcinoma-A multi-centre study in Sweden, Brazil and Romania. *Oral diseases*, *27*(2), 183–192. https://doi.org/10.1111/odi.13510

Tovaru, S., Costache, M., Perlea, P., Caramida, M., Totan, C., Warnakulasuriya, S., & Parlatescu, I. (2023). Oral leukoplakia: A clinicopathological study and malignant transformation. *Oral diseases*, *29*(4), 1454–1463. https://doi.org/10.1111/odi.14123

Tsai, K. Y., Su, C. C., Chiang, C. T., Tseng, Y. T., & Lian, I. B. (2017). Environmental heavy metal as a potential risk factor for the progression of oral potentially malignant disorders in central Taiwan. *Cancer epidemiology*, *47*, 118–124. https://doi.org/10.1016/j.canep.2017.02.003

Waldron, C. A., & Shafer, W. G. (1975). Leukoplakia revisited. A clinicopathologic study 3256 oral leukoplakias. *Cancer*, *36*(4), 1386–1392. https://doi.org/10.1002/1097-0142(197510)36:4<1386::aid-cncr2820360430>3.0.co;2-7

Wang, T. Y., Chiu, Y. W., Chen, Y. T., Wang, Y. H., Yu, H. C., Yu, C. H., & Chang, Y. C. (2018). Malignant transformation of Taiwanese patients with oral leukoplakia: A nationwide population-based retrospective cohort study. *Journal of the Formosan Medical Association = Taiwan yi zhi*, *117*(5), 374–380.

https://doi.org/10.1016/j.jfma.2018.01.017

Wang, Y. Y., Tail, Y. H., Wang, W. C., Chen, C. Y., Kao, Y. H., Chen, Y. K., & Chen, C. H. (2014). Malignant transformation in 5071 southern Taiwanese patients with potentially malignant oral mucosal disorders. *BMC oral health*, *14*, 99. https://doi.org/10.1186/1472-6831-14-99

Warnakulasuriya, S., Kovacevic, T., Madden, P., Coupland, V. H., Sperandio, M., Odell, E., & Møller, H. (2011). Factors predicting malignant transformation in oral potentially malignant disorders among patients accrued over a 10-year period in South East England. *Journal of oral pathology & medicine : official publication of the International Association of Oral Pathologists and the American Academy of Oral Pathology*, *40*(9), 677–683. https://doi.org/10.1111/j.1600-0714.2011.01054.x

Watabe, Y., Nomura, T., Onda, T., Yakushiji, T., Yamamoto, N., Ohata, H., ... Shibahara, T. (2016). Malignant transformation of oral leukoplakia with a focus on low-grade dysplasia. *Journal of Oral and Maxillofacial Surgery, Medicine, and Pathology*, *28*(1), 26–29. https://doi.org/10.1016/j.ajoms.2015.02.007

Wu, W., Wang, Z., & Zhou, Z. (2019). Risk Factors Associated With Malignant Transformation in Patients With Oral Leukoplakia in a Chinese Population: A Retrospective Study. *Journal of oral and maxillofacial surgery : official journal of the American Association of Oral and Maxillofacial Surgeons*, *77*(12), 2483–2493. https://doi.org/10.1016/j.joms.2019.08.002

Wu, X., Wang, R., Jiao, J., Li, S., Yu, J., Yin, Z., Zhou, L., & Gong, Z. (2018). Transglutaminase 3 contributes to malignant transformation of oral leukoplakia to cancer. *The international journal of biochemistry & cell biology*, *104*, 34–42. https://doi.org/10.1016/j.biocel.2018.08.016

Zhang, X., Kim, K. Y., Zheng, Z., Bazarsad, S., & Kim, J. (2017). Nomogram for risk prediction of malignant transformation in oral leukoplakia patients using combined biomarkers. *Oral oncology*, *72*, 132–139. https://doi.org/10.1016/j.oraloncology.2017.07.015

7. List of excluded studies with reasons (List S2)

7.1 Lack of essential data (n=6)

Becker, A. S., Holm, M., Liese, J., Engel, N., & Zimpfer, A. H. (2024). Diagnosis of differentiated dysplasia as a variant of oral epithelial dysplasia. *Oral diseases*, 10.1111/odi.14846. Advance online publication. https://doi.org/10.1111/odi.14846

Cai, X., Zhang, J., Li, L., Liu, L., Tang, M., Zhou, X., Peng, C., Li, X., Chen, X., Xu, M., Zhang, H., Wang, J., Huang, Y., & Li, T. (2024). Copy Number Alterations Predict Development of OSCC from Oral Leukoplakia. *Journal of dental research*, *103*(2), 138–146. https://doi.org/10.1177/00220345231217160

Gopinath, D., Thannikunnath, B. V., & Neermunda, S. F. (2016). Prevalence of Carcinomatous Foci in Oral Leukoplakia: A Clinicopathologic Study of 546 Indian Samples. *Journal of clinical and diagnostic research : JCDR*, *10*(8), ZC78–ZC83. https://doi.org/10.7860/JCDR/2016/16815.8305

Rushiti, A., Castellani, C., Cerrato, A., Fedrigo, M., Sbricoli, L., Bressan, E., Angelini, A., & Bacci, C. (2023). The Follow-Up Necessity in Human Papilloma Virus-Positive vs. Human Papilloma Virus-Negative Oral Mucosal Lesions: A Retrospective Study. *Journal of clinical medicine*, *13*(1), 58. https://doi.org/10.3390/jcm13010058

Wils, L. J., Poell, J. B., Brink, A., Evren, I., Brouns, E. R., de Visscher, J. G. A. M., Bloemena, E., & Brakenhoff, R. H. (2023). Elucidating the Genetic Landscape of Oral Leukoplakia to Predict Malignant Transformation. *Clinical cancer research : an official journal of the American Association for Cancer Research*, *29*(3), 602–613. https://doi.org/10.1158/1078-0432.CCR-22-2210

Yen, A. M., Chen, S. C., Chang, S. H., & Chen, T. H. (2008). The effect of betel quid and cigarette on multistate progression of oral pre-malignancy. *Journal of oral pathology & medicine : official publication of the International Association of Oral Pathologists and the American Academy of Oral Pathology*, *37*(7), 417–422. https://doi.org/10.1111/j.1600-0714.2008.00652.x

7.2 Overlapping population (n=8)

Pindborg, J. J., Mehta, F. S., & Daftary, D. K. (1975). Incidence of oral cancer among 30,000 villagers in india in a 7-year follow-up study of oral precancerous lesions. *Community dentistry and oral epidemiology*, *3*(2), 86–88. https://doi.org/10.1111/j.1600-0528.1975.tb00286.x

Serban, A. E., Nicolae, C., Parlatescu, I., & Tovaru, S. (2023). Oral Leukoplakia. A Five-Year Follow-Up Study. *Maedica*, *18*(4), 645–650. https://doi.org/10.26574/maedica.2023.18.4.645

Silverman S., Jr (1968). Observations on the clinical characteristics and natural history of oral leukoplakia. *Journal of the American Dental Association (1939)*, *76*(4), 772–777. https://doi.org/10.14219/jada.archive.1968.0141

Sugar, L., and Banoczy, J.(1950). Untersuchungen bei Praekanzerose der Mundschleimhaut. *Deutsch Zahn Mund Kieferheilk, 30, 132.*

Wang, T., Wang, L., Yang, H., Lu, H., Zhang, J., Li, N., & Guo, C. B. (2019). Development and validation of nomogram for prediction of malignant transformation in oral leukoplakia: A large-scale cohort study. *Journal of oral pathology & medicine : official publication of the International Association of Oral Pathologists and the American Academy of Oral Pathology*, *48*(6), 491–498. https://doi.org/10.1111/jop.12862

Wils, L. J., Poell, J. B., Peferoen, L. A. N., Evren, I., Brouns, E. R., de Visscher, J. G. A. M., van der Meij, E. H., Brakenhoff, R. H., & Bloemena, E. (2023). The role of differentiated dysplasia in the prediction of malignant transformation of oral leukoplakia. *Journal of oral pathology & medicine : official publication of the International Association of Oral Pathologists and the American Academy of Oral Pathology*, *52*(10), 930–938. https://doi.org/10.1111/jop.13483

Yao, Y. L., Wang, Y. F., Li, C. X., Wu, L., & Tang, G. Y. (2022). Management of oral leukoplakia by ablative fractional laser-assisted photodynamic therapy: A 3-year retrospective study of 48 patients. *Lasers in surgery and medicine*, *54*(5), 682–687. https://doi.org/10.1002/lsm.23534

Zhang, X., Kim, K. Y., Zheng, Z., Kim, H. S., Cha, I. H., & Yook, J. I. (2017). Snail and Axin2 expression predict the malignant transformation of oral leukoplakia. *Oral oncology*, *73*, 48–55. https://doi.org/10.1016/j.oraloncology.2017.08.004

7.3 Other study design (n=4)

Barfi Qasrdashti, A., Habashi, M. S., Arasteh, P., Torabi Ardakani, M., Abdoli, Z., & Eghbali, S. S. (2017). Malignant Transformation in Leukoplakia and Its Associated Factors in Southern Iran: A Hospital Based Experience. *Iranian journal of public health*, *46*(8), 1110–1117.

Kudva, A., Kumar, M., John, E. R., & Dhara, V. (2023). Occurrence of Second Oral Potentially Malignant Disorder following Excision of Primary Lesion: A Prospective Study of Cases from a Tertiary Care Centre. *Journal of maxillofacial and oral surgery*, *22*(1), 252–257. https://doi.org/10.1007/s12663-022-01764-9

Li, J., Liu, Y., Zhang, H., & Hua, H. (2020). Association between hyperglycemia and the malignant transformation of oral leukoplakia in China. *Oral diseases*, *26*(7), 1402–1413. https://doi.org/10.1111/odi.13372

Mustafa, M. B., Hassan, M. O., Alhussein, A., Mamoun, E., El Sheikh, M., & Suleiman, A. M. (2019). Oral leukoplakia in the Sudan: clinicopathological features and risk factors. *International dental journal*, *69*(6), 428–435. https://doi.org/10.1111/idj.12509

7.4 Trial/interventionist (n=10)

Brouns, E. R., Baart, J. A., Karagozoglu, K. H., Aartman, I. H., Bloemena, E., & van der Waal, I. (2013). Treatment results of CO2 laser vaporisation in a cohort of 35 patients with oral leukoplakia. *Oral diseases*, *19*(2), 212–216. https://doi.org/10.1111/odi.12007

Bukovszky, B., Fodor, J., Tóth, E., Kocsis, Z. S., Oberna, F., Ferenczi, Ö., & Polgár, C. (2023). Malignant Transformation and Long-Term Outcome of Oral and Laryngeal Leukoplakia. *Journal of clinical medicine*, *12*(13), 4255. https://doi.org/10.3390/jcm12134255

Campos, W. G., Esteves, C. V., Gallo, C. B., Domaneschi, C., Aranha, A. C. C., & Lemos, C. A. (2022). Treatment of oral leukoplakia with CO2 laser (10,600 nm): analysis of 37 cases. *Brazilian oral research*, *36*, e014. https://doi.org/10.1590/1807-3107bor-2022.vol36.0014

Del Corso, G., Gissi, D. B., Tarsitano, A., Costabile, E., Marchetti, C., Montebugnoli, L., & Foschini, M. P. (2015). Laser evaporation versus laser excision of oral leukoplakia: A retrospective study with long-term follow-up. *Journal of cranio-maxillo-facial surgery : official publication of the European Association for Cranio-Maxillo-Facial Surgery*, *43*(6), 763–768. https://doi.org/10.1016/j.jcms.2015.04.009

Georgaki, M., Avgoustidis, D., Theofilou, V. I., Piperi, E., Pettas, E., Kalyvas, D. G., Vlachodimitropoulos, D., Perisanidis, C., Lazaris, A. C., & Nikitakis, N. G. (2021). Recurrence in Oral Premalignancy: Clinicopathologic and Immunohistochemical Analysis. *Diagnostics (Basel, Switzerland)*, *11*(5), 872. https://doi.org/10.3390/diagnostics11050872

Lee, J. J., Hong, W. K., Hittelman, W. N., Mao, L., Lotan, R., Shin, D. M., Benner, S. E., Xu, X. C., Lee, J. S., Papadimitrakopoulou, V. M., Geyer, C., Perez, C., Martin, J. W., El-Naggar, A. K., & Lippman, S. M. (2000). Predicting cancer development in oral leukoplakia: ten years of translational research. *Clinical cancer research : an official journal of the American Association for Cancer Research*, *6*(5), 1702–1710.

Mogedas-Vegara, A., Hueto-Madrid, J. A., Chimenos-Küstner, E., & Bescós-Atín, C. (2015). The treatment of oral leukoplakia with the CO2 laser: A retrospective study of 65

patients. *Journal of cranio-maxillo-facial surgery : official publication of the European Association for Cranio-Maxillo-Facial Surgery*, *43*(5), 677–681. https://doi.org/10.1016/j.jcms.2015.03.011

Monteiro, L., Barbieri, C., Warnakulasuriya, S., Martins, M., Salazar, F., Pacheco, J. J., Vescovi, P., & Meleti, M. (2017). Type of surgical treatment and recurrence of oral leukoplakia: A retrospective clinical study. *Medicina oral, patologia oral y cirugia bucal*, *22*(5), e520–e526. https://doi.org/10.4317/medoral.21645

Yang, S. W., Lee, Y. S., Wu, P. W., Chang, L. C., & Hwang, C. C. (2021). A Retrospective Cohort Study of Oral Leukoplakia in Female Patients-Analysis of Risk Factors Related to Treatment Outcomes. *International journal of environmental research and public health*, *18*(16), 8319. https://doi.org/10.3390/ijerph18168319

Zhou, B., Yuan, K. F., & Chen, W. L. (2021). Use of allograft dermal matrix for repairing large oral epithelial defects: Outcomes of patients with lingual and buccal leukoplakia. *Journal of cosmetic dermatology*, *20*(9), 2753–2757. https://doi.org/10.1111/jocd.13973

7.5 Meeting abstract(n=2)

Weisberger D. (1957). Precancerous lesions. *Journal of the American Dental Association (1939)*, *54*(4), 507–508. https://doi.org/10.14219/jada.archive.1957.0065

Roed-Petersen B. (1971). Cancer development in oral leukoplakia: Followup of 331 patients (Abstr). *J Dent Res, 50, 71*

7.6 Other sites and OPMDs associated (n=3)

Archibald, H., Kalland, K., Kuehne, A., Ondrey, F., Roby, B., & Jakubowski, L. (2023). Oral Premalignant and Malignant Lesions in Fanconi Anemia Patients. *The Laryngoscope*, *133*(7), 1745–1748. https://doi.org/10.1002/lary.30370

McCord, C., Achita, P., Kiss, A., Magalhaes, M. A., Darling, M., & Bradley, G. (2023). Progression to malignancy in oral potentially malignant disorders: a retrospective study of 5,036 patients in Ontario, Canada. *Oral surgery, oral medicine, oral pathology and oral radiology*, *136*(4), 466–477. https://doi.org/10.1016/j.oooo.2023.06.006

Redman, R. S., Diehl, S. R., Jones-Richardson, T., Silva, R. G., Yeh, C. K., Malley, K. J., Farish, S. E., Duffy, M. B., Craig, R. M., & Winn, D. M. (2023). Follow-up study of veterans with white and red oral mucosal lesions at Veterans Affairs Dental Clinics. *Clinical and experimental dental research*, *9*(1), 82–92. https://doi.org/10.1002/cre2.677

7.7 Report not retrieved (n=1)

Skach, M.; Svoboda, O., and Kubat, K.(1960). Prispevek k problemu leukoplakie. Acta Univ Carol Ser Med Suppl, 10, 363.
